# Rewiring of Glucose and Lipid Metabolism Induced by G Protein-Coupled Receptor 17 Silencing Enables the Transition of Oligodendrocyte Progenitors to Myelinating Cells

**DOI:** 10.3390/cells11152369

**Published:** 2022-08-02

**Authors:** Davide Marangon, Matteo Audano, Silvia Pedretti, Marta Fumagalli, Nico Mitro, Davide Lecca, Donatella Caruso, Maria P. Abbracchio

**Affiliations:** 1 Department of Pharmaceutical Sciences, Università degli Studi di Milano, 20133 Milan, Italy; davide.marangon@unimi.it (D.M.); davide.lecca@unimi.it (D.L.); 2 Department of Pharmacological and Biomolecular Sciences, Università degli Studi di Milano, 20133 Milan, Italy; matteo.audano@unimi.it (M.A.); silvia.pedretti@unimi.it (S.P.); marta.fumagalli@unimi.it (M.F.); nico.mitro@unimi.it (N.M.); donatella.caruso@unimi.it (D.C.)

**Keywords:** oligodendrocyte progenitor cell, oligodendrocyte, energy metabolism, glycolysis, lactate, myelin lipids, transcriptomics, metabolomics, lipidomics, myelination

## Abstract

In the mature central nervous system (CNS), oligodendrocytes (OLs) provide support and insulation to axons thanks to the production of a myelin sheath. During their maturation to myelinating cells, OLs require energy and building blocks for lipids, which implies a great investment of energy fuels and molecular sources of carbon. The oligodendroglial G protein-coupled receptor 17 (GPR17) has emerged as a key player in OL maturation; it reaches maximal expression in pre-OLs, but then it has to be internalized to allow terminal maturation. In this study, we aim at elucidating the role of physiological GPR17 downregulation in OL metabolism by applying transcriptomics, metabolomics and lipidomics on differentiating OLs. After GPR17 silencing, we found a significant increase in mature OL markers and alteration of several genes involved in glucose metabolism and lipid biosynthesis. We also observed an increased release of lactate, which is partially responsible for the maturation boost induced by GPR17 downregulation. Concomitantly, GPR17 depletion also changed the kinetics of specific myelin lipid classes. Globally, this study unveils a functional link between GPR17 expression, lactate release and myelin composition, and suggests that innovative interventions targeting GPR17 may help to foster endogenous myelination in demyelinating diseases.

## 1. Introduction

In the central nervous system (CNS), oligodendrocytes (OLs) are the cells responsible for the production of myelin, an insulating structure characterized by an exceptionally high lipid content, that preserves axonal function and enables fast neuronal conduction [1]. These specialized functions require that oligodendrocyte precursor cells (OPCs) undergo a tightly orchestrated differentiation program, during which they progressively change their metabolism and modify the composition of their plasma membrane by synthesizing the typical myelin lipids [2]. One OL can myelinate up to 50 axons, with as many as 150 layers of compacted myelin membrane per segment, thus requiring a substantial energetic demand [3]. In vitro studies have shown that the rate of glycolysis versus oxidative phosphorylation depends on the OL differentiation stage [4,5], and suggested that newly generated OLs/late OPCs are the most vulnerable to complex I inhibition [6]. Under optimal conditions, cultured postnatal OPCs and OLs rely on oxidative phosphorylation for ATP synthesis, while adult OLs preferentially use glycolysis [7]. Interestingly, in the presence of a low glucose environment, OL development and myelination can be supported by monocarboxylates, such as ketone bodies and lactate, whose trafficking into/out of the cell requires monocarboxylate transporters (MCTs). Of note, Ichihara and colleagues showed that lactate was able to rescue OPC cycling and differentiation under low glucose conditions, and that this effect was inhibited by a lactate transporter inhibitor, suggesting that lactate availability allows OLs to sustain oxidative phosphorylation and myelin lipid synthesis [4]. Different classes of lipids are present in OL and myelin membranes, including cholesterol, phospholipids and glycosphingolipids. Although there are no lipid species uniquely existing in myelin, consistent changes in the proportion of several lipid species were described in OL membrane during differentiation [8].

OLs also play an active and dynamic role in neuronal metabolism [9]. They take up glucose coming from the bloodstream and perform glycolysis to produce pyruvate and lactate, which is then delivered to the axonal compartment through MCTs, thus enabling mitochondrial respiration and ATP generation [10].

During their differentiation, OPCs are influenced by extrinsic factors that dynamically modulate their functions through the interactions with specific receptors [11]. Among these, G protein-coupled receptor 17 (GPR17) has emerged as a promising remyelinating target in multiple sclerosis (MS). GPR17 is a receptor transiently expressed in OPCs, reaching its maximal levels in pre-OLs at intermediate differentiation stages expressing the immature O4 marker. Its transient expression is necessary for a timely and physiological OPC maturation; indeed, after the O4-positive stage, this receptor has to be progressively downregulated, to allow terminal maturation and myelination [12,13]. Any interference with this timely and physiological expression results in dysfunction and, eventually, in myelination failure. Overexpression of GPR17 has been described in several models of disease such as brain ischemia [14], cuprizone- and lysolecithin-induced demyelination [15,16], Alzheimer’s-like conditions [17], amyotrophic lateral sclerosis [18] and experimental autoimmune encephalomyelitis (EAE) [15,19]. Marked GPR17 upregulation and/or accumulation of GPR17-expressing cells at the border of demyelinated lesions has been also observed in patients affected by MS [19,20], traumatic brain injury [21] and congenital leukoencephalopathy [22]. In the presence of strong pro-inflammatory conditions, GPR17-expressing cells accumulate at lesion sites, remaining stuck at intermediate stages and not able to contribute to remyelination, suggesting that dysregulation of receptor downregulation contributes to remyelination failure and disease progression. Despite these data, the molecular events consequent to GPR17 downregulation have not been fully elucidated. Of note, metabolic dysfunctions are thought to play an important role in the mechanism of progression of various neurodegenerative disorders [23], including MS [24,25,26]. The presence of metabolic dysfunction in MS demands the need to better understand the role of bioenergetic metabolites and their associated mechanisms in regulating OL function. Based on these considerations, in the present study, we aim at understanding whether and how GPR17 modulation directly affects OL bioenergetics during differentiation by means of transcript, metabolite and lipid profiling. Transcriptomic analysis showed that GPR17 silencing in differentiating OPCs increases the expression of mature markers and induces changes in several genes involved in the transcriptional regulation of lipid and glucose homeostasis. Moreover, our metabolomic analysis showed that, after GPR17 downregulation, OLs rewire their metabolism and increase the release of lactate, which can directly affect the OL differentiation program. Furthermore, by means of lipidomic analysis, we also showed that GPR17 silencing alters the abundance of myelin specific lipids. Together these findings suggest that GPR17 downregulation in differentiating OLs induces changes of both energy metabolism and membrane lipids required for their differentiation into mature myelinating cells, and that GPR17 targeting can be exploited to rescue metabolic dysfunctions typically associated to demyelinating diseases.

## 2. Materials and Methods

### 2.1. Animal Care

International (European law Dir. 2010/63/UE) and national (Italian law DL n. 26, 4 March 2014) guidelines for the care and use of animals were followed. All the procedures were approved by the Italian Ministry of Health (5247B.N.L2Y to M.P.A.). Pregnant rats were purchased from Charles River Italia and housed under a 12-h light/12-h dark cycle at 21 °C, with diet and water ad libitum, and environmental enrichment.

### 2.2. Rat OPC Isolation

Primary rat OPCs were obtained from postnatal day 2 (P2) Sprague–Dawley rats’ cerebral cortices pooled together as previously described [27,28]. OPCs were plated onto poly-D,L-ornithine-coated (final concentration 50 µg/mL; Sigma-Aldrich, Milan, Italy) 10 cm dishes (2–4 × 10^5^ cells/coverslip) for metabolomic analysis, onto poly-D,L-ornithine-coated 13-mm glass coverslips for immunocytochemistry (1–2 × 10^4^ cells/coverslip), and onto poly-D,L-ornithine-coated 6-well plates (7 × 10^4^ cells/coverslip) for lipidomic and qRT-PCR analysis. Cells were plated in Neurobasal medium supplemented with 2% B27 (Life Technologies, Monza, Italy), 2 mM L-glutamine, 10 ng/mL human platelet-derived growth factor BB (Sigma-Aldrich, Milan, Italy), and 10 ng/mL human basic fibroblast growth factor (Life Technologies, Monza, Italy) to promote proliferation for 3 days.

### 2.3. siRNA Transfection

OPCs were transfected immediately after switching from proliferating to differentiating medium (Neurobasal medium supplemented with 2% B27 (Life Technologies, Monza, Italy), 2 mM L-glutamine and 15 nM T3 hormone). On-target plus SMARTpool siRNA specific for rat GPR17 transcript (Dharmacon, Lafayette, CO, USA) were transfected at the final concentration of 50 nM with Lipofectamine RNAiMAX reagent (Life Technologies, Monza, Italy), following the manufacturer’s protocol. The transfection of a non-targeting RNA pool was used as a negative control.

### 2.4. Transcriptome Profiling and Microarray Analysis

RNA extraction and transcriptome analysis have been performed according to published protocols [27]. Briefly, OPCs were lysed with RLT buffer (Qiagen, Milan, Italy) 2 days after siRNA transfection and total RNA was extracted by means of RNeasy Micro kit (Qiagen, Milan, Italy), following the manufacturer’s protocol. RNA quality was assessed with Agilent 2100 Bioanalyzer (Agilent Technologies, Milan, Italy). RNA with RNA integrity number (RIN) > 7 was used for microarray analyses. Labelled cRNA was synthesized from 100 ng of total RNA using the Low Input Quick-Amp Labelling Kit, one colour (Agilent Technologies, Milan, Italy) in the presence of cyanine 3-CTP. Total RNA was hybridized on SurePrint G3 Rat Gene Expression Microarrays (#G4858A-074036, Agilent Technologies, Milan, Italy). This microarray consists of 60-mer DNA probes synthesized in situ, which represent 30,584 rat transcripts. Hybridization was performed at 65 °C for 17 h in a rotating oven (Microarray Facility, Laboratorio per le Tecnologie delle Terapie Avanzate, LTTA, Ferrara, Italy). One-color gene expression analysis was performed according to manufacturer’s procedure. Feature Extraction 10.7.3 software (Agilent Technologies, Milan, Italy) was used to obtain microarray raw data. A fold change of ±1.5 and an FDR-adjusted *p*-value < 0.07 were considered to obtain the list of genes differentially expressed between the two experimental conditions.

### 2.5. Bioinformatic Analysis

The software Metacore™ (Clarivate Analytics, London, UK) and QIAGEN’s Ingenuity^®^ Pathway Analysis (IPA^®^, QIAGEN Redwood City, www.qiagen.com/ingenuity, accessed on 14 May 2017) were used to perform a pathway-based clusterization on differentially expressed genes after GPR17 silencing in OPCs, to identify common biological processes. The ToppGene Suite was used to perform gene ontology-based enrichment analysis [29].

### 2.6. Targeted Metabolomics and Lipidomics

Metabolomic data were obtained by liquid chromatography coupled to tandem mass spectrometry (LC-MS/MS), as previously described [30,31], with some modifications described below. We used an API-3500 triple quadrupole mass spectrometer (AB Sciex, Framingham, MA, USA) coupled with an ExionLC™ AC System (AB Sciex). OPC cells from different time points and or transfected with siRNA were smashed in a tissue lyser for 1 min at maximum speed in 250 µL of ice-cold methanol/water/acetonitrile 55:25:20. Lysates were spun at 15,000× *g* for 15 min at 4 °C. Samples were then dried under N2 flow at 40 °C and resuspended in 125 µL of ice-cold MeOH/H_2_0/ACN 55:25:20 for subsequent analyses. Amino acid quantification was performed through previous derivatization. Briefly, 25 µL out of 125 µL of samples were collected and dried separately under N2 flow at 40 °C. Dried residues were resuspended in 50 µL of phenyl-isothiocyanate (PITC), EtOH, pyridine and water 5%:31.5%:31.5%:31.5%, and then incubated for 20 min at RT, dried under N2 flow at 40 °C for 90 min and finally resuspended in 100 µL of 5 mM ammonium acetate in MeOH/H_2_O 50:50. Quantification of different amino acids was performed by using a C18 column (Biocrates, Innsbruck, Austria) maintained at 50 °C. The mobile phases for positive ion mode analysis were phase A: 0.2% formic acid in water and phase B: 0.2% formic acid in acetonitrile. The gradient was T0: 100%A, T5.5: 5%A, T7: 100%A with a flow rate of 500 µL/min. Quantification of energy metabolites and cofactors was performed by using a cyano-phase LUNA column (50 mm × 4.6 mm, 5 µm; Phenomenex) by a 5.5 min run in negative ion mode with two separated runs. Protocol A; mobile phase A was water and phase B was 2 mM ammonium acetate in MeOH and the gradient was T0: 80%B 0.5 mL/min; T1: 80%B 0.5 mL/min; T1.01: 20%B 0.5 mL/min; T2.0: 20%B 0.5 mL/min; T2.01: 80%B 0.5 mL/min; T5.5: 80%B 0.5 mL/min. Protocol B; mobile phase A was water and phase B was 2 mM ammonium acetate in MeOH and the gradient was T0: 50%B 0.5 mL/min; T1: 50%B 0.5 mL/min; T1.01: 0%B 0.5 mL/min; T2.0: 0%B 0.5 mL/min; T2.01: 50%B 0.5 mL/min; T5.5: 50%B 0.5 mL/min. Acyl-carnitines quantification was performed on the same samples by using a C18 column (Biocrates, Innsbruck, Austria) maintained at 50 °C. Samples were analyzed by a 5.5 min run in positive ion mode. Mobile phases were A: 0.1% formic acid in H20 B: 0.1% formic acid in MeOH and the gradient was T0:30%B, T0.8: 30%A, T2: 100%B, T3: 100%B, T3.01: 30%B, T5.5: 30%B with a flow rate of 500 µL/min.

The levels of phospholipids and fatty acids were evaluated by means of (LC)-MS/MS according to published protocol [32,33] with some modifications described below. Phospholipids were analyzed in both negative and positive ionization on MeOH/H20/ACN extracts through an API-4000 triple quadrupole mass spectrometer (AB Sciex, Framingham, MA, USA) coupled with a HPLC system (Agilent) and CTC-PAL HTS autosampler (PAL System). For negative ionization analysis, lipids were separated through a cyano-phase LUNA column (50 mm × 4.6 mm, 5 µm; Phenomenex) in a 5 min run, and the mobile phase was 5 mM ammonium acetate in MeOH. For the positive ionization, analytes were separated on a XTerra RP18 3.5 µm 4.6mm × 100 mm Column (Waters) in an 8 min run, and the mobile phase was 0.1% formic acid in MeOH. Free fatty acids were analyzed in negative ion mode on a Hypersil Gold C8 column (Life Technologies) with a mobile phase consisting of 10 mM diisopropylethylamine and 15 mM acetic acid in H_2_O/MeOH 97:3. All metabolites analyzed in the described protocols were previously validated with pure standards, except for phospholipids, where only those representatives of the different classes were validated. MultiQuant™ software (version 3.0.3, AB Sciex) was used for data analysis and peak review of chromatograms. For data processing, raw areas were normalized by the median of all metabolite areas in the same sample. Specifically, we defined the relative metabolite abundance ($m_{a}^{N}$) as:

$$m_{a}^{N}=\frac{Xn}{M_{a=1}^{n}a}$$

where *Xn* represents the peak area of metabolite n for samples a, b, …, z, and $M_{a=1}^{n}a$ represents the median of peak areas of metabolite n for samples *a, b, …, z*. Obtained data were then transformed by log10-transformation and Pareto scaled to correct for heteroscedasticity, reduce the skewness of the data and reduce mask effects [34]. In detail, obtained values were transformed by log10 and then scaled by Pareto’s method as follows:

$${\bar{x}}_{ij}=\frac{x_{ij}-{\bar{x}}_{i}}{\sqrt{s_{i}}}$$

where x_ij_ is the transformed value in the data matrix (i (metabolites), j (samples)) and s_i_ is the standard deviation of transformed metabolite values [35]. Obtained values were considered as relative metabolite levels. Data processing and analysis were performed by MetaboAnalyst 5.0 web tool [36].

### 2.7. Immunocytochemistry and Cell Counting

Cells were fixed in a 4% paraformaldehyde phosphate-buffered solution containing 4% sucrose. The following primary antibodies were used: rabbit anti-GPR17 (1:100; Cayman Chemical), rat anti-MBP (1:200; Merck Millipore, Milan, Italy). Incubation of primary antibodies were performed for 2.5 h at room temperature or overnight at 4 °C. Cells were then incubated for 1 h at room temperature with secondary antibodies conjugated to either AlexaFluor 488 or AlexaFluor 555 (1:600; Life Technologies, Monza, Italy). All the antibodies were diluted in a phosphate-buffered blocking solution (pH 7.4) containing 0.3% Triton X-100. Nuclei were labeled with the UV fluorescent dye Hoechst 33258 (1:10,000; Life Technologies, Monza, Italy). Coverslips were then mounted in a fluorescent mounting medium (Dako). Positive cells for the selected markers were counted from 20 random fields for each coverslip (0.07 mm^2^/field). The result was expressed as a percentage over the number of nuclei, and then normalized versus controls set to 100%.

### 2.8. Treatment with AR-C155858

One day after siRNA transfection (day in differentiation 1), control and GPR17-silenced OPCs were treated with AR-C155858 (Tocris, UK; final concentration: 1 μM) or vehicle (0.04% DMSO). After two days (day in differentiation 3), OPCs were lysed for subsequent RNA analysis or treated again and then collected at day in differentiation 5.

### 2.9. Total RNA Extraction, Retrotranscription and qPCR Analysis

Cells were lysed with Trizol reagent (Life Technologies, Monza, Italy) at 1, 2, 3 or 5 days after transfection. Total RNA was extracted by using a Direct-zol RNA micro-prep kit (Zymo Research, Germany). For gene expression analysis, cDNA synthesis was performed starting from 400–800 ng of total RNA using SensiFAST™ cDNA synthesis kit (Bioline, London, UK). The expression of all genes was analysed with SensiFAST™ SYBR Supermix (Bioline, London, UK) and normalized to GAPDH expression using the CFX96 real time PCR system (Bio-rad, Milan, Italy) following the manufacturer’s protocol.

### 2.10. Statistical Analysis

Data are presented as mean ± SEM and analyzed with the GraphPad Prism 8.0.1 software. For all comparisons between two groups with a normal distribution, two-tailed unpaired Student t-tests were performed. Two-tailed paired Student t-tests were used for analysis on human samples. For multiple comparison of groups with a normal distribution, one-way or two-way analysis of variance (ANOVA) accompanied by Tukey’s or Sidak’s multiple post hoc test were used. Unless otherwise specified, *p* < 0.05 was considered as statistically significant.

## 3. Results

### 3.1. GPR17 Silencing in Differentiating OPCs Significantly Altered the Expression of Genes Involved in Lipid and Glucose Metabolism

To highlight the biological processes significantly affected by GPR17 expression during OL differentiation, we interfered with its expression by transfecting rat OPCs with SMARTpool siRNAs specific for rat GPR17. After having validated successful GPR17 knockdown by qRT-PCR (Figure S1), we performed a microarray analysis of 30,584 rat transcripts in the GPR17-silenced versus control OPCs and analyzed the differentially expressed genes (DEGs) by different bioinformatic tools. First, the 640 DEGs have been analyzed by MetaCore, to perform a pathway enrichment analysis and identify the processes predicted to be significantly affected. This analysis suggested alteration of the mTOR signaling, which has been already shown to be linked to GPR17 function [37], and of other processes known to be relevant for OPC maturation, such as “cytoskeleton remodeling” and “regulation of lipid metabolism” (Table 1).

**Figure 1:**
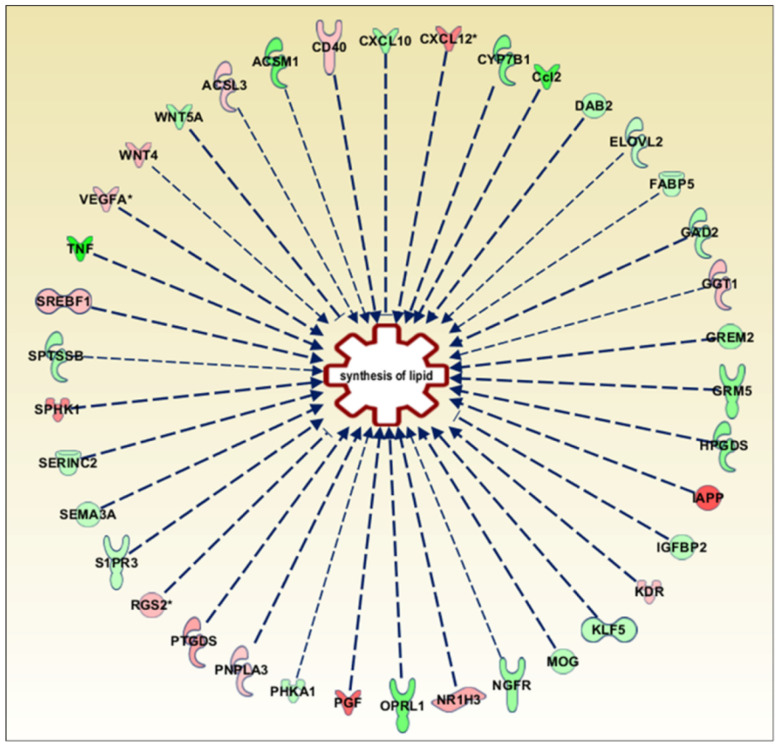
Ingenuity pathway analysis tool (IPA, Qiagen) was used to analyse dataset and explore the diseases and biological processes which are predicted to be increasing or decreasing based on the pattern of differentially expressed genes in the dataset. From this analysis we extrapolated the scheme in the Figure that includes the genes related to lipid synthesis differentially expressed in our dataset (*p*-value = 9.67 × 10^−4^). The full names of these genes, together with the relative fold change (log2 ratio) are reported in Table S1. In red, upregulated genes; in green, downregulated genes.

**Figure 2:**
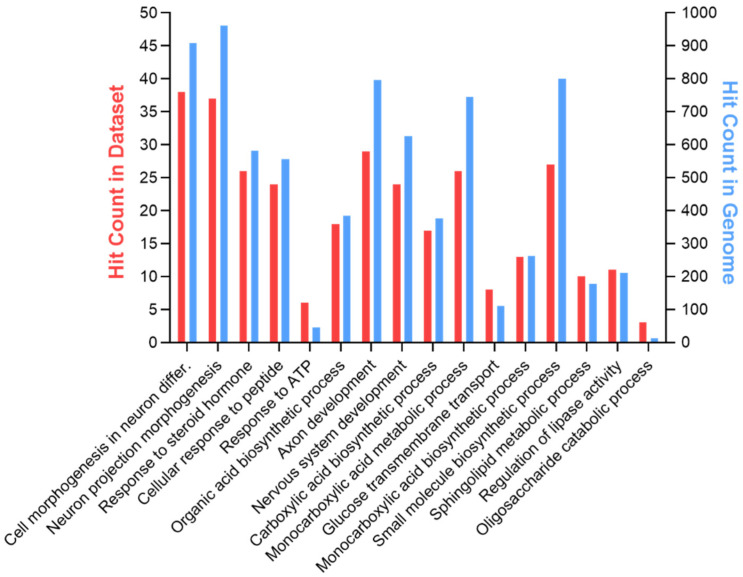
The Toppgene suite has been used to perform a GO-based enrichment analysis on the DEGs. The picture shows some of the significant biological processes related to the central nervous system and cell metabolism potentially altered by GPR17 silencing. Blue bars indicate the total genes associated to each term; red bars indicate the genes in common with our dataset for each term. The complete list of biological processes resulting from this analysis and the relative *p*-values are reported in Table S2.

Then, the Ingenuity pathway analysis (IPA) tool was used to perform an analysis match, which automatically identifies curated IPA datasets with significant similarities and differences compared to a query dataset. This analysis strengthens the predicted link between GPR17 expression and lipid metabolism, showing that the expression changes of 38 genes in our dataset (Table S1) is associated with altered fatty acid synthesis (Figure 1). Among these, GPR17 silencing induced upregulation of LXRα (liver X receptor alpha, NR1H3 in Figure 2 and Table S1, a nuclear receptor responding to oxysterols) and SREBP1 (sterol regulatory element binding protein 1, SREBF1 in Figure 1 and Table S1), two key players in fatty acid and cholesterol synthesis. In our dataset, we also observed that several altered genes, including PDH (pyruvate dehydrogenase), Ldha (lactate dehydrogenase alpha) and Pdk1 (pyruvate dehydrogenase kinase 1), are related to glucose metabolism and Krebs’ cycle, suggesting the potential involvement of the GPR17 receptor in the regulation of these metabolic processes. According to this, a gene ontology-based enrichment analysis (ToppGene suite) on the DEGs revealed alteration of “monocarboxylic acid metabolic process” (GO:0032787, *p*-value: 0.02), beyond other processes related to CNS development and cell metabolism, such as “regulation of nervous system development” (GO:0051960, *p*-value: 0.033) and “sphingolipid metabolic processes” (GO:0006665, *p*-value: 0.043) (Figure 2; full list in Table S2).

Globally, these changes suggest that GPR17 silencing may act as a trigger to address cells to myelination, by tuning several genes involved in the transcriptional regulation of lipid and glucose homeostasis, as well as in the utilization of cholesterol and lipids for myelin production.

### 3.2. Metabolomic Analysis during OPC Differentiation In Vitro

**Figure 3:**
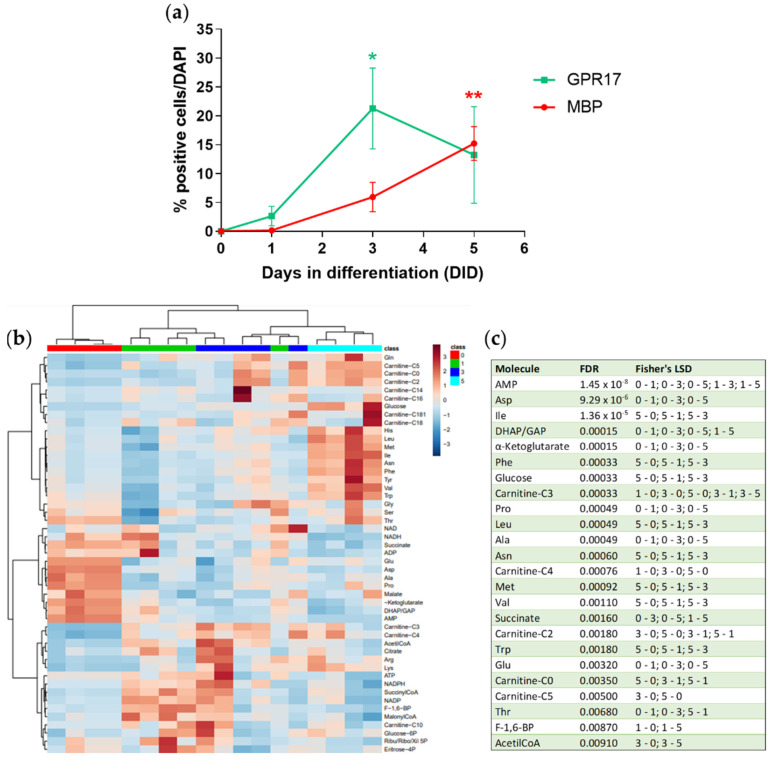
(**a**) The graph shows changes in the percentage of GPR17^+^ and MBP^+^ cells during differentiation. *n* = 3 per group; one-way ANOVA with Tukey’s test. * *p* < 0.05, ** *p* < 0.01) (**b**) The heatmap shows metabolite abundance in each single sample stopped at 0, 1, 3 or 5 DID. The abundance of the analyzed metabolites is represented by a chromatic scale ranging from dark blue (very low abundance) to dark brown (very high abundance). *n* = 4–5 per each group. (**c**) Metabolites with an abundance significantly different among the conditions (0, 1, 3 and 5 days in differentiation, DID; one-way ANOVA with Fisher’s LSD test).

To assess the correlation between GPR17 expression and the activation of specific metabolic pathways, we performed a metabolomic analysis of cultured OPCs during their physiological maturation. OPCs were maintained in differentiation medium and then lysed at four different time points (after 0, 1, 3, and 5 days in differentiation, DID), that correspond to different stages of OL maturation. In parallel, to assess that in all the experiments maturation occurred with the expected timing, OPCs were cultured and stained for GPR17 and MBP. The results showed that the number of GPR17-expressing cells reached its maximum at 3 DID and then returned towards basal levels at 5 DID, whereas the number of MBP-positive cells progressively increased during maturation (Figure 3a), consistently with previous results [37]. OPC metabolomic evaluation was performed by liquid chromatography tandem mass spectrometry (LC-MS/MS) as previously described [38], focusing on energetic metabolites involved in glycolysis, the pentose phosphate pathway, the Krebs cycle, fatty acid β-oxidation, their cofactors NADH, NAD^+^, NADPH, NADP^+^, ATP, ADP, AMP, amino acids and some of their derivatives. The metabolomic profiles of the four time points analyzed are reported in Figure 3b. At day 0, the levels of several metabolites belonging and/or linked to the Krebs cycle (e.g., succinate, malate, alpha-Ketoglutarate, glutamate, aspartate, alanine, and proline) were higher, as expected from cells coming from a highly proliferating state (Figure 3c). At intermediate stages (from DID 1 to 3), OPCs showed a marked upregulation of molecules involved in fatty acid and cholesterol synthesis. Accordingly, we observed increased levels of acetylCoA (Figure 3c), that represents the precursor of cholesterol and fatty acids and of malonylCoA, a specific intermediate of fatty acid synthesis. At DID 5, we observed increased levels of several acyl-carnitines and amino acids in parallel to a significant increase of free intracellular glucose (Figure 3c). We also observed decreased levels of AMP and ADP from day 0 to DID 5 and a transient increase of ATP between DID 1 and DID 3. These data indicate that, from day 0 to DID 3, corresponding to the temporal window during which GPR17 reaches its maximum expression, OPCs mainly used glucose and amino acids to sustain cholesterol and fatty acid biosynthesis. At DID 5, as highlighted from increased acyl-carnitine levels, OPCs likely rely on fatty acid utilization.

Together, these data indicate that during physiological differentiation OPCs progressively rewire their energetic metabolism to become mature myelinating OLs.

### 3.3. Metabolomic Analysis after GPR17 Silencing during OPC Maturation In Vitro

To unveil the contribution of the GPR17 receptor in driving the energy metabolic changes occurring during cell differentiation, we performed metabolomics on GPR17-silenced OPCs, in comparison to control OPCs receiving a scrambled RNA. We first examined the impact of GPR17 silencing on OPC maturation by immunofluorescence staining for GPR17 and MBP.

**Figure 4:**
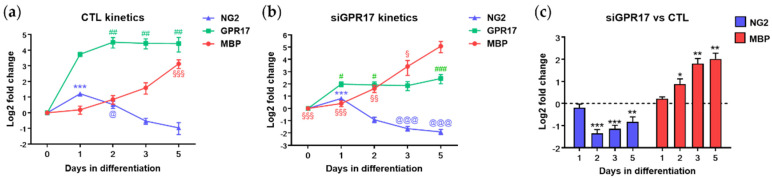
Effect of GPR17 silencing on OPC maturation. The expression levels of GPR17 (green), NG2 (blue) and MBP (red) were evaluated by real-time PCR during differentiation in control conditions (**a**), cells transfected with scramble RNA and after GPR17 silencing (**b**), and normalized versus expression at T0 (set to 0). *n* = 6 per each time point. One-way ANOVA with Tukey’s multiple comparisons test. (**a**): *** *p* < 0.001 vs. DID 0-2-3-5, @ *p* < 0.05 vs. DID 3-5; ## *p* < 0.01 vs. day 0; §§§ *p* < 0.001 vs. DID 0-1-2-3; (**b**): *** *p* < 0.001 vs. DID 0-2-3-5, @@@ *p* < 0.001 vs. day 0, # *p* < 0.05, ### *p* < 0.001 vs. day 0, § *p* < 0.05, §§ *p* < 0.01, §§§ *p* < 0.001 vs. DID 5. (**c**) NG2 and MBP expression in GPR17-silenced OPCs vs. control OPCs (set to 0) at each time point. Fold change (FC) has been reported as LOG_2_(FC). One sample t-test, * *p* < 0.05; ** *p* < 0.01; *** *p* < 0.001 vs. relative DID in control OPCs.

As expected, after receptor silencing, we found a reduction in GPR17 staining, clearly visible at 3 DID. We also observed a marked increase in the number of mature MBP-positive OLs at both 3 DID (not shown) and 5 DID (Figure S2). In parallel, a detailed qPCR analysis was performed at five time points (0, 1, 2, 3 and 5 days in differentiation, DID), revealing, in control OPCs, a strong increase in GPR17 expression after 1 DID, that reaches significant levels starting after 2 DID, a progressive increase in MBP expression up to 5 DID and a significant decrease in the early marker NG2 (Figure 4a). Conversely, after GPR17 silencing (Figure 4b), GPR17 mRNA reached lower levels at DID1 and then remained stably low, MBP expression further increased and NG2 further decreased. A direct comparison of NG2 and MBP expression in GPR17-silenced versus control OPCs at each time point is shown in Figure 4c. Taken together, these results suggest that, under these experimental conditions, GPR17 silencing in OPCs accelerated their differentiation process. Then, OPC metabolomics was performed by LC-MS/MS at the five selected timepoints. Comparison of metabolomic data obtained from GPR17-silenced and control OPCs at each time point allowed us to identify the metabolites that are significantly affected by GPR17 silencing. The list of the analyzed metabolites, together with the relative fold changes and *p*-values, are reported in Table S3.

**Figure 5:**
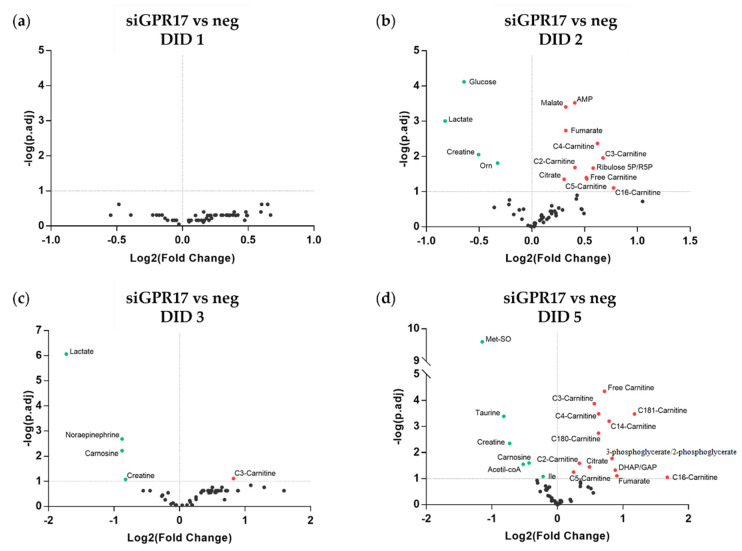
Metabolomic changes induced by GPR17 silencing in OPCs. (**a**–**d**) The abundance of each metabolite after GPR17 silencing has been normalized to that in the relative control sample. The resulting fold changes were used to generate a volcano plot for each time point (1, 2, 3, 5 days in differentiation). The points over the dotted horizontal line represent the metabolites that have shown a statistically significant change (Fisher’s LSD corrected by FDR; *p.adj* < 0.1). In green, metabolites with lower expression, in red, those with higher expression, after GPR17 silencing. *n* = 7–9 per each group. DID: days in differentiation.

At DID 1 (Figure 5a), we did not find significant changes. At DID 2 (Figure 5b), we found a significant increase in some metabolites of the Krebs cycle (malate, fumarate, citrate), a modest increase in free carnitine (C0), acetyl-carnitine(C2), propionyl-carnitine (C3:0), butyryl-carnitine (C4:0), valeryl-carnitine (C5:0), hexadecanoyl-carnitine (C16:0) and a significant reduction in glucose, lactate, creatine, and ornithine. The reduction of lactate and creatine and the increase of propionyl-carnitine (C3) was also observed at DID 3 (Figure 5c). At DID 5 (Figure 5d), we still found an increase in free carnitine (C0), acetyl-carnitine (C2), propionyl-carnitine (C3:0), butyryl-carnitine (C4:0), valeryl-carnitine (C5:0), tetradecanoyl-carnitine (C14:0), hexadecanoyl-carnitine (C16:0), octadecanoyl-carnitine (C18:0), octadecenoyl-carnitine (C18:1), similarly to what originally observed in more differentiated cells (see Figure 3), an increase in dihydroxyacetone-3-phosphate/glyceraldehyde-3-phosphate (DHAP/GAP), fumarate and citrate, and a decrease in acetyl-CoA, Met-SO, creatine, taurine and carnosine.

### 3.4. GPR17 Modulates Lactate Release during OPC Maturation

**Figure 6:**
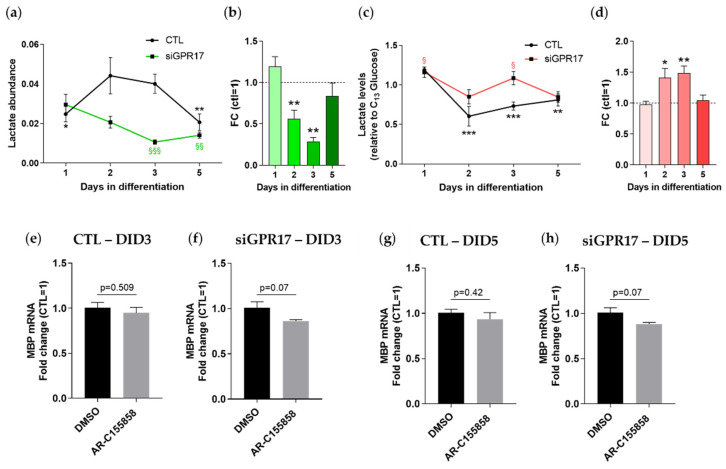
GPR17 silencing in OPCs alters lactate metabolism. (**a**) Lactate abundance during OPC maturation in control conditions (green line) and after GPR17 silencing (orange line). *n* = 7–9 per each group. One-way ANOVA with Tukey’s multiple comparisons test. * *p* < 0.05, ** *p* < 0.01 vs. DID2 CTL; §§ *p* < 0.01, §§§ *p* < 0.001 vs. DID1 siGPR17 (**b**) Lactate levels after GPR17 silencing were normalized versus control samples (set to 1) at each time point (*n* = 7–9). ** *p* < 0.01; one sample t-test. Conditioned media from GPR17-silenced and control OPCs were collected from the same culture used for metabolomic analysis. Lactate levels in the media were evaluated by LC-MS/MS. (**c**) Abundance of lactate in the extracellular space during OPC maturation in control conditions (green line) and after GPR17 silencing (orange line). One-way ANOVA with Tukey’s multiple comparisons test. ** *p* < 0.01, *** *p* < 0.001 vs. DID1 CTL; § *p* < 0.05 vs. DID5 siGPR17. (**d**) Extracellular lactate levels after GPR17 silencing were normalized versus control samples (set to 1) at each time point. * *p* < 0.05; ** *p* < 0.01; one sample *t*-test. The effect of MCT1 inhibition in control (**e**,**g**) and GPR17-silenced (**f**,**h**) OPCs has been evaluated by analyzing the expression of MBP, in inhibitor-treated samples vs. relative controls (vehicle treated), at at 3 and 5 days of differentiation. *n* = 6 per each group.

Based on the metabolomics results described above, GPR17 silencing was found to reduce the intracellular levels of lactate. To assess if this reduction reflects a general decrease in lactate metabolism or rather an increased release of this metabolite, we measured its levels both in cell lysates and in the corresponding media at different timepoints. As shown in Figure 6, in control conditions, intracellular lactate reached the highest abundance at DID 2 (Figure 6a) (when GPR17 receptor expression is also maximal), and then it reduced when the cells became mature. Instead, after GPR17 silencing, the rise of intracellular lactate was abolished, and its levels further decreased toward differentiation. Indeed, comparison of lactate levels in GPR17-silenced OPCs versus control OPCs at each time points highlighted a reduction in its levels at DID 2 and 3 (Figure 6b). The extracellular lactate followed different kinetics, showing significant reduction compared to DID 1 at DID 2-3-5 in control conditions, whereas in GPR17-silenced OPCs it remained stably higher and then significantly decreased at DID 5 (Figure 6c). However, when comparing lactate levels at the different timepoints between the two experimental conditions, GPR17 silencing hampered the physiological reduction, increasing lactate presence in the medium at DID 2 and 3 (Figure 6d), suggesting an increased release.

One of the mechanisms by which OPCs can release lactate is through the monocarboxylate transporter MCT1. Moreover, recent studies reported that OPCs themselves can acquire extracellular lactate through MCT receptors promoting their cell cycling and differentiation [39]. Thus, we wondered whether the increased release of lactate induced by GPR17 silencing could be mediated by MCT1 and whether this may be partially responsible for the effect observed on OPC maturation in the same conditions (Figure 4). To this aim, we treated OPCs with AR-C155858, a selective inhibitor of MCT1, during normal differentiation and after GPR17 silencing. As expected, lactate release was successfully inhibited in both conditions (Figure S3).

The effect of MCT1 inhibition on OPC maturation in the different experimental conditions was evaluated by analyzing the expression levels of MBP. A modest reduction in the gene expression of MBP, which is close to statistical significance (*p* = 0.07), has been found only in samples transfected with specific siRNA for GPR17 (Figure 6e–h); however, no significant variation emerged in the samples transfected with control RNA (siNEG). These results suggest that the increase in extracellular lactate mediated by GPR17 downregulation fosters maturation of OPCs; however, the small extent of MBP reduction indicates that this metabolite is not the only factor involved in the process.

### 3.5. Lipidomic Analysis after GPR17 Silencing during OPC Maturation

**Figure 7:**
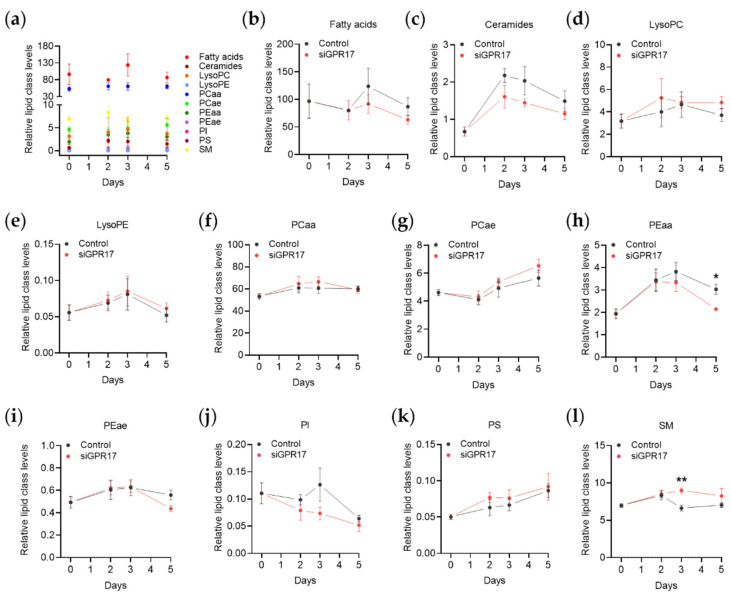
Changes in lipidic classes abundance induced by GPR17 silencing during OPC differentiation. The abundance of each lipid class in control conditions at different time points is reported (**a**). The kinetics of each lipid class after GPR17 silencing were analysed: (**b**) fatty acids, (**c**) ceramides, (**d**) LysoPC (lysophosphatidyl-cholines), (**e**) LysoPE (lysophosphatidyl-ethanolamines), (**f**) PCaa (diacyl-phosphatidyl-cholines), (**g**) PCae (acyl-alkyl-phosphatidyl-cholines), (**h**) PEaa (di-acyl-phosphatidyl-ethanolamines), (**i**) PEae (acyl-alkyl-phosphatidyl-ethanolamines), (**j**) PI (phosphatidyl-inositols), (**k**) PS (phosphatidyl-serines), (**l**) SM (sphingomyelins). *n* = 5 for each group. Kinetics: Two-way ANOVA followed by Tukey’s multiple comparison test. siGPR17: Two-way ANOVA followed by Sidak’s multiple comparison test. * *p.adj* < 0.05; ** *p.adj* < 0.01.

The results of the transcriptomic analysis after GPR17 silencing also highlighted a potential link between its function and the synthetic pathways of lipids and cholesterol, which are the main components of myelin. To further investigate the role of GPR17 in lipid metabolism, we applied LC-MS/MS to the lipidomic analysis of GPR17-silenced OPCs after 2, 3 and 5 days in differentiation, compared to day 0. The list of the analyzed lipids, together with the relative fold changes and *p*-values, are reported in Table S4. The two most abundant lipid classes in the experimental groups were fatty acids and the diacyl-phosphatidylcholine (PCaa) family. During differentiation of control OPCs, we did not find any significant changes in the abundance of any lipid class. Instead, GPR17-silenced OPCs showed a trend toward altered abundance of free fatty acids, ceramides, acyl-alkyl-phosphatidylcholine (PCae) and phosphatidylinositol (PI) compared to control OPCs, especially at DID 3 and 5, but only diacyl-phosphatidylethanolamine (PEaa) and sphingomyelin (SM) showed a statistically significant change (Figure 7).

**Figure 8:**
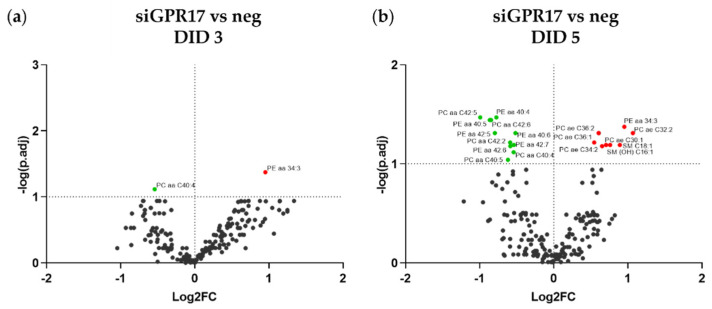
Lipidomic changes induced by GPR17 silencing in OPCs. (**a**,**b**) The abundance of each lipid after GPR17 silencing has been normalized to that in the relative control sample. The resulting fold changes were used to generate a volcano plot for each time point (3, 5 days in differentiation). The points over the dotted horizontal line represent the metabolites that have shown a statistically significant change (Fisher’s LSD corrected by FDR; *p.adj* < 0.1). In green, lipids with lower expression, in red, those with higher expression, after GPR17 silencing. *n* = 5 per each group.

Comparison of lipidomic data obtained from GPR17-silenced and control OPCs at each time point allowed us to identify the lipids that were significantly affected by GPR17 silencing. After 2 DID, no significant changes were found (data not shown). After 3 DID, diacyl-phosphatidylcholine (PCaa) C40:4 was less abundant and diacyl-phosphatidylethanolamine (PEaa) C34:3 was more abundant in GPR17-silenced OPCs (Figure 8a). After 5 days in differentiation, GPR17 silencing led to several changes in lipid abundance: reduction of several diacyl-PCs and diacyl-PE and a concomitant increase in several plasmalogens (acyl-alkyl-PC) along with sphingomyelin (SM) C18:1 and SM(OH) C16:1 (Figure 8b). These data indicate that the down regulation of GPR17 alters the lipid profile of the main component of myelin as PC and PE.

## 4. Discussion

In recent years, several studies have highlighted the relevance of GPR17 in OL differentiation, validating this receptor not only as a marker of a precise maturation time window (i.e., the O4^+^ pre-OL intermediate stage), but demonstrating that it also acts as a key regulator of OPC differentiation [12,19]. Notably, during physiological differentiation, after OPC intermediate stages, GPR17 has to be silenced to allow cells’ terminal maturation, an event that likely occurs through both downregulation/internalization of membrane receptor complexes and nuclear repression of gpr17 gene transcription [37,40]. Defective GPR17 silencing, i.e., any conditions leading to persistent GPR17 aberrant expression (such as those observed in several neurodegenerative conditions associated to demyelination), blocks cells at immature stages and contributes to remyelination defects [15,41]. This has led to the hypothesis that the timely GPR17 silencing acts as a “green signal” to promote effective myelination by favoring metabolic and biochemical processes necessary for cell maturation [13]. However, the nature and precise sequela of the events activated by GPR17 silencing are still unknown. Here, we addressed this gap by, first, performing a whole transcriptomic analysis on OPCs under normal conditions and after mimicking GPR17 downregulation by siRNA technology, followed by analysis of the dataset of differentially expressed genes by pathway- and ontology-based approaches. This initial analysis suggested alterations of various pathways and biological processes known for their importance in the maturation of OLs, including the mTOR and Wnt signaling pathways, cytoskeleton remodeling, and regulation of energy and OPC metabolism.

Targeted metabolomic analysis during OPC differentiation and following GPR17 silencing allowed us to get insights on the possible link between GPR17 expression and OPC metabolism. In early OPCs, when GPR17 expression is still physiologically low, cells displayed high levels of metabolites belonging to the Krebs cycle, which were much less abundant at later times, indicating their consumption to produce other key metabolites. This is in line with literature data showing that OPCs are more metabolically active than OLs, and that ATP production in rodent OPCs is predominantly dependent on oxidative phosphorylation [7]. At intermediate stages (from DID 1 to DID 3), pre-OLs showed a marked upregulation of molecules involved in fatty acid and cholesterol synthesis, like acetylCoA (that represents the precursor of cholesterol and fatty acids) and malonylCoA (a specific intermediate of fatty acid synthesis). These changes perfectly coincided with the timing of GPR17 expression in OPCs, which is very low at day 0, when OPCs are induced to exit the cell cycle and start differentiation, and gradually reaches its maximal expression at DID 3, when pre-OLs have already downregulated metabolites involved in the cell cycle and are upregulating molecules related to fatty acid synthesis. Globally, these data indicate that when GPR17 reaches its maximal expression, pre-OLs mainly use glucose and amino acids to sustain cholesterol and fatty acid biosynthesis. Of note, it is known that, despite aerobic glycolysis generating ATP with less efficiency than oxidative phosphorylation, it produces the carbon chain precursors necessary to support protein and lipid biosynthesis for myelin production [42], limits the production of ROS, and promotes the production of long-lived lipids required by OL myelin [7]. We also observed decreased levels of AMP and ADP from day 0 to DID 5 and a transient increase of ATP between DID 1 and 3. These data are fully compatible with GPR17 expression and its consolidated role in regulating (in concert with other GPCRs) the intracellular production of cyclic AMP (cAMP) from ATP during OL differentiation [13,43,44]. When GPR17 expression starts rising (DID 1 to 3), endogenous ligands promote a gradual increase of the activity of the receptor-coupled Gi protein, that, in turn, reduces the production of cAMP [12], thus resulting in increased intracellular levels of ATP (here acting as the substrate of adenylyl cyclase for cAMP synthesis), concomitantly with the increased levels of lipid biosynthesis metabolites (acetylCoA and malonylCoA). These data are also in line with the concept that anabolic metabolism (such as lipid synthesis) usually occurs in a favorable energy status.

After GPR17 silencing, results from our metabolomic analysis suggest an extensive rewiring of OPC metabolism. We observed changes in the abundance of intermediates of the Krebs cycle, such as fumarate, malate, and citrate (the starting substrate for the synthesis of lipids and cholesterol) and of other metabolites described in the literature for their critical role in OL survival and differentiation, as well as in myelin synthesis, such as creatine, taurine and lactate [39,45,46]. Despite these alterations all being potentially interesting, we decided to focus our analyses on the role of lactate, based on previous data implicating it in OL cellular metabolism, differentiation and myelination [4,47], as well as in communication with nearby neurons and glia [48]. Analysis of intracellular lactate abundance during OPC physiological differentiation showed kinetics following that of GPR17 (high levels at intermediate stages, low levels during the initial and final stages), whereas after GPR17 silencing, lactate levels decreased prematurely, reaching a minimum at intermediate stages. The relationship between GPR17 expression and lactate abundance has previously been observed in recent studies, where an increase in glycolysis and lactate production, secondary to the alteration of the cAMP-PKA-PDK1 axis, has been found in OLs deriving from GPR17 knock-out animals [49]. According to this, our microarray analysis on GPR17-silenced OPCs demonstrated the upregulation of genes associated to glycolysis and lactate production (PFK, PDK1, LDH); however, on the other hand, our metabolic data showed a reduction in lactate intracellular levels. To shed light on these apparently contrasting results, we evaluated the release of this metabolite in the culture media. Increased lactate levels were found at the same times at which lowered lactate concentrations were found in cells’ cytoplasm, suggesting release of this metabolite extracellularly, and that GPR17 in OLs could play an important role in the transport of energy metabolites to neuronal cells. This hypothesis is corroborated by a recent finding showing that, in GPR17 knockout mice, increased lactate levels in the environment of hypothalamic neurons can modulate neuronal activity through activation of the AKT and STAT3 pathways [49]. Based on this evidence, we speculate that a similar mechanism could apply to taurine and creatine, which are known to positively regulate the myelination process [45,46], and whose intracellular levels are indeed reduced in GPR17-silenced OPCs. Of note, it has been shown that neurons do not express the enzymes responsible for the synthesis of these metabolites, suggesting that they can only derive from neighboring glial cells [50,51].

The ability of OLs to import lactate or release it extracellularly is mediated by monocarboxylate transporter 1 (MCT1), responsible for the passive transport of lactate and hydrogen towards the cytoplasm or the extracellular environment in response to its concentrations in the two compartments [47,52]. Despite in vivo observations indicating that the lack of MCT1 in OLs and their progenitors in the early post-natal period and in early adulthood does not affect myelination, possibly due to the availability of other energy intermediates such as glucose, during adulthood, loss of MCT1 leads to axonopathy and hypo-myelination, demonstrating the essential role that monocarboxylic acids play in the energetic homeostasis of OLs [53]. In our study, the increase in extracellular lactate observed after GPR17 silencing was associated to accelerated maturation, raising the possibility that lactate released by mature OLs can be taken up by immature OLs to enhance their maturation in an autocrine fashion. To assess this hypothesis, we blocked lactate release by exposing GPR17-silenced OPCs to a selective MCT1 inhibitor. Reduction of lactate efflux indeed resulted in decreased OPC maturation, suggesting that GPR17 downregulation could foster release of lactate extracellularly, thus propagating the maturation process to nearby cells. Accordingly, recent studies have shown that lactate can directly promote the cell cycling rate and differentiation of OPCs [39].

The expression changes observed after GPR17 silencing also suggested a potential link between GPR17 expression and the activation of lipid and cholesterol synthetic pathways. Among the most relevant changes, we found an increased expression of LXRα and its target genes, SREBP1c, ABCG1 and ABCA1. The ability of LXRα to promote cell differentiation in OLs has been demonstrated in vivo following lysolecithin-induced demyelination: LXRα activation by both natural (i.e., oxysterols) and synthetic agonists promoted remyelination of areas affected by demyelinating injury [54]. Oxysterols are also pro-inflammatory molecules capable of activating several G protein-coupled receptors, including GPR17 [55], whose ability to respond to emergency signals related to oxidative stress, neuroinflammation and neurodegeneration is widely known. Expression changes of the transcription factors described above are also necessary for the reorganization of lipid metabolism that occurs during the maturation of OLs [56], allowing these cells to increase the synthesis of specific lipid classes and gradually change the composition of their plasma membrane [2]. This shift characterizes the transition from OPCs to mature OLs; for example, it has been shown that some myelin-specific glycosphingolipids start to be produced in the proximity of terminal differentiation and are maintained in mature myelinating OLs [57]. Our lipidomic data suggest that GPR17 expression may directly influence the abundance of myelin-specific lipids, such as ceramides and sphingomyelins. The correct balance between synthesis and degradation of sphingolipids has been shown to play a critical role in maintaining myelin integrity [58,59]. Moreover, we detected that GPR17 knockdown at DID 5 led to decreased levels of several diacyl-PCs and diacyl-Pes, which are the main species represented in myelin membrane as reported in the literature [60,61,62]. The finding that these lipids may play a role in stabilizing the intraperiod line of myelin [61] further supports the key role of GPR17 as a key player in determining myelin composition and function. Despite a decreased levels of diacyl-PCs and diacyl-PEs, we also found increased levels of several plasmalogens (i.e., acyl-alkyl-PC) upon GPR17 depletion at DID5. These PC-derived plasmalogens have been proposed as protective agents in myelin, since they prevent membrane lipid peroxidation by reactive oxygen species [63,64]. The protecting effects of plasmalogens has been ascribed to the hydrogen atom adjacent to the vinyl ether bond. Indeed, this hydrogen atom can be more susceptible to oxidation with respect to the ester linkage present in diacyl-PCs and diacyl-PEs [63,64]. Furthermore, myelin plasmalogens participate in membrane formation and/or in the maintenance [65,66]. Based on our results, we infer that GPR17 is also important in regulating the balance between diacyl-PC and diacyl-PE and their respective plasmalogens to ensure the optimal structure and function of myelin. However, it should be considered that plasmalogens might be increased in the absence of GPR17 as a protective mechanism given their antioxidant role. Alteration in myelin lipids has been also described in diabetic rats [62], underlying a link between glucose metabolism and myelination. It has been reported that diseases characterized by dysregulation of lipid or glucose metabolism (i.e., dyslipidaemias and diabetes) are linked to higher MS risk [67,68], even though the exact pathogenetic mechanisms linking these metabolic dysfunctions to MS are still largely debated. Moreover, mass spectrometric analysis revealed an increased abundance of phospholipids and a reduction in sphingolipid content in the normal appearing white matter (NAWM) of subjects with active MS [69], suggesting that specific metabolic alterations may appear before the onset of demyelinating lesions. Of note, in a previous study, we have shown that the NAWM of MS patients presents the highest abundance of GPR17-expressing cells compared to MS lesions and to the WM of healthy subjects [20].

Our study is the first showing the involvement of GPR17 downregulation in rewiring OPC metabolism during differentiation, but some limitations/caveats should be taken into account. First, in this study we used primary cultures, which in comparison to cell lines have both advantages and disadvantages. Despite primary OLs being more prone to reach the myelinating stage, data obtained using these cells may have higher variability, due to the fact that OPCs are not synchronized and therefore they follow slightly different maturation kinetics in vitro between independent experiments. However, to tackle this issue, we applied a method of analysis that identifies samples deviating in their metabolomic profile from other samples of the same group, allowing the reallocation to the proper group. Second, we used a targeted metabolomic approach that allowed us to gather several metabolite levels and changes in representative metabolic pathways. As such, a comprehensive untargeted metabolomic analysis (with no a priori definition of metabolites to be detected) can be further informative of other metabolites not considered in our targeted metabolomics, thus generating novel working hypotheses in the field. Third, although our study represents a significant advance in understanding the role of GPR17 in OPC maturation, further work remains necessary to assess the relevance of the metabolic changes induced by its downregulation in pathological conditions. In this context, future studies will be aimed at assessing whether GPR17 modulation can be exploited to improve OL metabolism during demyelination/remyelination dynamics in animal models of MS and whether GPR17 dysregulation may correlate with specific metabolic alterations in human MS lesions and in the NAWM.

Taken together, our results suggest that the GPR17 receptor may act as a checkpoint necessary to inhibit precocious myelination and maintain cells’ reactivity to extracellular signals and highlight the involvement of GPR17 downregulation in the coordination of energy and lipid metabolism rearrangements required for OPC maturation, providing important hints on the bioactive metabolites and lipids regulated by the receptor. Based on our previous data on alterations of GPR17 expression in MS pathogenesis and remyelination failure, we suggest that these functional and mechanistic insights may inspire new pharmacological and therapeutic approaches for the cure of neurodegenerative diseases.

## Figures and Tables

**Table 1:** The software MetaCore has been used to perform a pathway enrichment analysis on the DEGs after GPR17 silencing. Table shows the most significant pathways resulting from the analysis, the number of associated genes and of common genes included in the dataset.

Pathways	Total Genes	*p*-Value	FDR	Genes in Dataset
mTORC1 downstream signaling	61	6.05 × 10^−5^	2.50 × 10^−2^	7
TGF, WNT and cytoskeleton remodelling	111	8.73 × 10^−5^	2.50 × 10^−2^	9
IL-6 signaling in multiple myeloma	51	1.80 × 10^−4^	3.27 × 10^−2^	6
Regulation of lipid metabolism	19	2.29 × 10^−4^	3.27 × 10^−2^	4
Regulation of endothelial progenitor cell differentiation from adult stem cells	60	4.42 × 10^−4^	4.87 × 10^−2^	6
Regulation of lung epithelial progenitor cell differentiation	41	5.34 × 10^−4^	4.87 × 10^−2^	5
Regulation of tissue factor signaling in cancer	43	6.68 × 10^−4^	4.87 × 10^−2^	5
Role of IL-8 in angiogenesis	65	6.81 × 10^−4^	4.87 × 10^−2^	6

## Data Availability

Datasets and raw data are publicly available in GEO Profile (GEO ID: GSE208409).

## Supplementary Materials

The following supporting information can be downloaded at: https://www.mdpi.com/article/10.3390/cells11152369/s1, Figure S1: GPR17 silencing with specific siRNAs in maturating OPCs; Table S1: Up- and downregulation of key genes involved in lipid synthesis induced by GPR17 silencing; Table S2. GO-based enrichment analysis on DEGs after GPR17 silencing in OPCs; Figure S2: Effect of GPR17 silencing on OPC maturation; Table S3. Descriptive tables of metabolomic analysis. Figure S3: Effect of AR-C155858 on lactate release; Table S4. Descriptive tables of lipidomic analysis.
