# Supplementary material for: Rewiring of Glucose and Lipid Metabolism Induced by G Protein-Coupled Receptor 17 Silencing Enables the Transition of Oligodendrocyte Progenitors to Myelinating Cells
Source: Cells. 2022 Aug 2;11(15):2369. doi: 10.3390/cells11152369 (PMC9368002; doi:10.3390/cells11152369)
Supplement: Supplementary file 1 [file cells-11-02369-s001.zip › cells-1821884-supplementary.pdf]

Rewiring of glucose and lipid metabolism induced by G protein-coupled receptor 17 silencing enables the transition of oligodendrocyte progenitors to myelinating cells

Davide Marangon[1], Matteo Audano[2], Silvia Pedretti[2], Marta Fumagalli[2], Nico Mitro[2], Davide Lecca[1], Donatella Caruso[2], Maria P. Abbracchio[1,*]

[1] Department of Pharmaceutical Sciences, Università degli Studi di Milano; davide.marangon@unimi.it (D.M.); davide.lecca@unimi.it (D.L.)
[2] Department of Pharmacological and Biomolecular Sciences, Università degli Studi di Milano; matteo.audano@unimi.it (M.A.); silvia.pedretti@unimi.it (S.P.); marta.fumagalli@unimi.it (M.F.); nico.mitro@unimi.it (N.M.); donatella.caruso@unimi.it (D.C.)
* Correspondence: mariapia.abbracchio@unimi.it; Tel.: +390250318304

Supplementary Material

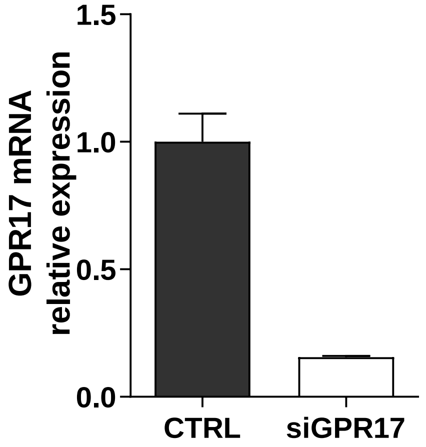

Figure S1. GPR17 silencing with specific siRNAs. Oligodendrocyte precursor cells (OPCs) were plated in presence of growth factors (bFGF and PDGF) for 3 days to promote proliferation. Then, cells were switched to differentiating medium, and simultaneously transfected with a pool of specific GPR17 siRNAs (or scramble siRNAs for control cells) to obliterate GPR17 expression. OPCs were lysed 48 hours after transfection for subsequent RNA extraction and microarray analysis. In these samples, GPR17 expression was reduced to 20% as determined by qRT-PCR. Data are presented as mean ± SD from 2 independent experiments.

Table S1. Up- and down-regulation of key genes involved in lipid synthesis induced by GPR17 silencing.

Gene Symbol	Gene name	Refseq	LOG2 FC
Acsl3	Acyl-CoA synthetase long-chain family member 3	NM_057107	1.51
Acsm1	Acyl-CoA synthetase medium-chain family member 1	NM_001108502	-2.71
Ccl2	Chemokine (C-C motif) ligand 2	NM_031530	-3.38
Cd40	CD40 molecule, TNF receptor superfamily member 5	XM_006235511	1.58
Cxcl10	Chemokine (C-X-C motif) ligand 10	NM_139089	-1.93
Cxcl12	Chemokine (C-X-C motif) ligand 12	NM_001033883	2.70
Cyp7b1	Cytochrome P450, family 7, subfamily b, polypeptide 1	NM_019138	-2.31
Dab2	Disabled 2, mitogen-responsive phosphoprotein	NM_024159	-1.66
Elovl2	ELOVL fatty acid elongase 2	NM_001109118	-1.51
Fabp5	Fatty acid binding protein 5, epidermal	NM_145878	-1.54
Gad2	Glutamate decarboxylase 2	NM_012563	-1.74
Ggt1	Gamma-glutamyltransferase 1	NM_053840	1.63
Grem2	Gremlin 2	NM_001105974	-1.85
Grm5	Glutamate receptor, metabotropic 5	NM_017012	-2.11
Hpgds	Hematopoietic prostaglandin D synthase	XM_006236602	-2.10
Iapp	Amylin	XM_006237614	3.75
Igfbp2	Insulin-like growth factor binding protein 2	NM_013122	-1.57
Kdr	Kinase insert domain receptor	NM_013062	1.60
Klf5	Kruppel-like factor 5	NM_053394	-1.55
Mog	Myelin oligodendrocyte glycoprotein	NM_022668	-1.59
Ngfr	Nerve growth factor receptor	NM_012610	-1.92
Nr1h3	Nuclear receptor subfamily 1, group H, member 3	NM_031627	1.92
Pgf	Placental growth factor	NM_053595	3.16
Phka	Phosphorylase kinase, alpha 1	XM_006257101	-1.55
Pnpla3	Patatin-like phospholipase domain containing 3	NM_001282324	1.50
Ptgds	Prostaglandin D2 synthase	NM_013015	1.99
Rgs2	Regulator of G-protein signaling 2	NM_053453	1.69
S1pr3	Sphingosine-1-phosphate receptor 3	NM_001271143	-1.53
Sema3a	Semaphorin 3A	XM_006235989	-1.51
Serinc2	Serine incorporator 2	NM_001031656	-1.55
Sphk1	Sphingosine kinase 1	NM_133386	2.49
Sptssb	Serine palmitoyltransferase, small subunit B	NM_001271299	-1.78
Srebf1	Sterol regulatory element binding transcription factor 1	NM_001276707	1.65
Tnf	Tumor necrosis factor	NM_012675	-4.54
Vegfa	Vascular endothelial growth factor A	NM_001287107	1.63
Wnt4	Wingless-type MMTV integration site family, member 4	NM_053402	1.77
Wnt5a	Wingless-type MMTV integration site family, member 5A	XM_008771007	-1.79

Table S2. GO-based enrichment analysis on DEGs after GPR17 silencing in OPCs. The Toppgene suite has been used to perform a GO-based enrichment analysis on the DEGs. P-value method: probability density function. Multiple correction method: false discovery rate (FDR) and the Benjamini-Hochberg (B&H) method. The table shows all the biological processes that reach the statistical significance and the associated genes (Hit Count in Dataset gene symbol).

ID	Name	q-value FDR B&H	Hit Count in Dataset	Hit Count in Genome	Hit Count in Dataset (gene symbol)
GO:0048667	cell morphogenesis involved in neuron differentiation	1.71E-03	38	909	PRMT8,RPS6KA5,LGR4,GRXCR1,LRTM2,WNT4,CHN1,SEMA3A,S100B,MYO3B,FGFR2,MMP14,PLS1,CXCL12,VEGFA,COL6A3,SEMA4B,WNT5A,PPFIA2,SLITRK3,CRMP1,SLITRK5,AUTS2,ATP2B2,SEMA3E,HECW1,SRRM4,FSTL4,GFRA2,NECTIN1,NGFR,CNTN2,SHANK1,NTRK3,GRB10,GRIN1,LZTS1,EPHA4
GO:0048754	branching morphogenesis of an epithelial tube	1.71E-03	16	210	LGR4,WNT4,ACVR1,PGF,SEMA3A,FGFR2,MMP14,CXCL12,VEGFA,WNT5A,CELSR1,SEMA3E,MYCN,KDR,TNF,CSMD1
GO:0090102	cochlea development	1.71E-03	9	64	OTOF,MYO3B,WNT5A,FRZB,SLC17A8,ATP2B2,GABRB2,NTRK3,EPHA4
GO:0034765	regulation of ion transmembrane transport	1.71E-03	31	687	ENPP1,KCNIP1,FABP5,CACNG3,SCN3A,CCL2,KCNK6,KCNK13,AZIN1,CNKSR3,AOC3,OCLN,PRSS8,HECW1,CXCL10,KCNB1,KCNC1,NFE2L2,KCNJ2,ABCC8,GLP1R,KCNQ3,CACNA1G,SHANK1,CACNA1C,GRB10,GRIN1,KCNG3,GRM5,RGS2,TNF
GO:0006935	chemotaxis	1.71E-03	36	870	RPS6KA5,LGR4,LRTM2,WNT4,LTB4R2,CHN1,PGF,SEMA3A,CCL2,CYP7B1,GPNMB,MMP14,CXCL12,VEGFA,COL6A3,SEMA4B,WNT5A,CXCL14,CRMP1,SEMA3E,CXCL10,SMOC2,GFRA2,CMTM8,NECTIN1,NGFR,KDR,CNTN2,CLXN,NTRK3,GRB10,CXCL1,LGALS3,CMTM7,CDH13,EPHA4
GO:0034762	regulation of transmembrane transport	1.71E-03	31	691	ENPP1,KCNIP1,FABP5,CACNG3,SCN3A,CCL2,KCNK6,KCNK13,AZIN1,CNKSR3,AOC3,OCLN,PRSS8,HECW1,CXCL10,KCNB1,KCNC1,NFE2L2,KCNJ2,ABCC8,GLP1R,KCNQ3,CACNA1G,SHANK1,CACNA1C,GRB10,GRIN1,KCNG3,GRM5,RGS2,TNF
GO:0048839	inner ear development	1.71E-03	21	363	GRXCR1,OTOF,MYO3B,FGFR2,PLS1,WNT5A,CXCL14,CELSR1,DACH2,FRZB,SLC17A8,ATP2B2,GABRB2,MYCN,BCR,MAFB,DLX3,NTRK3,GSC,ADGRG6,EPHA4
GO:0007169	transmembrane receptor protein tyrosine kinase signaling pathway	1.80E-03	36	880	EPS15,CRIM1,RPS6KA5,ENPP1,WNT4,CHN1,PGF,FZD4,TYRO3,AP3S1,CCL2,ATP6V1C2,FGFR2,VEGFA,WNT5A,TXNIP,IGFBP2,RASSF2,SREBF1,FSTL4,SMOC2,BCR,GFRA2,NGFR,KDR,NTRK3,GRB10,NR1H3,GRIN1,ARPC1B,EIF4EBP1,LTK,REPS2,HCK,CDH13,EPHA4
GO:0050918	positive chemotaxis	2.22E-03	10	90	PGF,GPNMB,CXCL12,VEGFA,WNT5A,CXCL10,KDR,NTRK3,LGALS3,CDH13
GO:0042330	taxis	2.22E-03	36	903	RPS6KA5,LGR4,LRTM2,WNT4,LTB4R2,CHN1,PGF,SEMA3A,CCL2,CYP7B1,GPNMB,MMP14,CXCL12,VEGFA,COL6A3,SEMA4B,WNT5A,CXCL14,CRMP1,SEMA3E,CXCL10,SMOC2,GFRA2,CMTM8,NECTIN1,NGFR,KDR,CNTN2,CLXN,NTRK3,GRB10,CXCL1,LGALS3,CMTM7,CDH13,EPHA4
GO:0048841	regulation of axon extension involved in axon guidance	2.22E-03	7	39	WNT4,SEMA3A,CXCL12,VEGFA,SEMA4B,WNT5A,SEMA3E

GO:0030335	positive regulation of cell migration	2.22E-03	30	684	ACVR1,PGF,SEMA3A,RIN2,CCL2,GPNMB,MMP14,CXCL12,VEGFA,ONE-CUT2,SEMA4B,WNT5A,CXCL14,FAM83H,MAPRE1,AOC3,SEMA3E,HDAC9,MYLK,CXCL10,DAB2,SMOC2,NFE2L2,SPHK1,KDR,NTRK3,GRIN1,LGALS3,TNF,CDH13
GO:0048812	neuron projection morphogenesis	2.74E-03	37	961	PRMT8,RPS6KA5,LGR4,LRTM2,WNT4,CHN1,RNF157,FZD4,SEMA3A,S100B,FGFR2,MMP14,CXCL12,VEGFA,COL6A3,SEMA4B,WNT5A,PPFIA2,SLI-TRK3,CRMP1,SLI-TRK5,AUTS2,SEMA3E,HECW1,SRRM4,FSTL4,GFRA2,NEC-TIN1,NGFR,KDR,CNTN2,SHANK1,NTRK3,GRB10,GRIN1,LZTS1,EPHA4
GO:0051272	positive regulation of cellular component movement	2.74E-03	31	733	ACVR1,PGF,SEMA3A,RIN2,CCL2,GPNMB,MMP14,CXCL12,VEGFA,ONE-CUT2,SEMA4B,WNT5A,CXCL14,FAM83H,MAPRE1,AOC3,SEMA3E,HDAC9,MYLK,CXCL10,DAB2,BCL6,SMOC2,NFE2L2,SPHK1,KDR,NTRK3,GRIN1,LGALS3,TNF,CDH13
GO:0043583	ear development	2.74E-03	21	393	GRXCR1,OTOF,MYO3B,FGFR2,PLS1,WNT5A,CXCL14,CELSR1,DACH2,FRZB,SLC17A8,ATP2B2,GABRB2,MYCN,BCR,MAFB,DLX3,NTRK3,GSC,ADGRG6,EPHA4
GO:0010721	negative regulation of cell development	2.74E-03	16	245	SEMA3A,VEGFA,SEMA4B,WNT5A,FRZB,SEMA3E,MYCN,FSTL4,BRINP1,ABCC8,NGFR,NPPC,NTRK3,S1PR3,TNF,EPHA4
GO:0061138	morphogenesis of a branching epithelium	2.86E-03	16	247	LGR4,WNT4,ACVR1,PGF,SEMA3A,FGFR2,MMP14,CXCL12,VEGFA,WNT5A,CELSR1,SEMA3E,MYCN,KDR,TNF,CSMD1
GO:0120039	plasma membrane bounded cell projection morphogenesis	3.15E-03	37	977	PRMT8,RPS6KA5,LGR4,LRTM2,WNT4,CHN1,RNF157,FZD4,SEMA3A,S100B,FGFR2,MMP14,CXCL12,VEGFA,COL6A3,SEMA4B,WNT5A,PPFIA2,SLI-TRK3,CRMP1,SLI-TRK5,AUTS2,SEMA3E,HECW1,SRRM4,FSTL4,GFRA2,NEC-TIN1,NGFR,KDR,CNTN2,SHANK1,NTRK3,GRB10,GRIN1,LZTS1,EPHA4
GO:2000147	positive regulation of cell motility	3.15E-03	30	713	ACVR1,PGF,SEMA3A,RIN2,CCL2,GPNMB,MMP14,CXCL12,VEGFA,ONE-CUT2,SEMA4B,WNT5A,CXCL14,FAM83H,MAPRE1,AOC3,SEMA3E,HDAC9,MYLK,CXCL10,DAB2,SMOC2,NFE2L2,SPHK1,KDR,NTRK3,GRIN1,LGALS3,TNF,CDH13
GO:0048858	cell projection morphogenesis	3.49E-03	37	984	PRMT8,RPS6KA5,LGR4,LRTM2,WNT4,CHN1,RNF157,FZD4,SEMA3A,S100B,FGFR2,MMP14,CXCL12,VEGFA,COL6A3,SEMA4B,WNT5A,PPFIA2,SLI-TRK3,CRMP1,SLI-TRK5,AUTS2,SEMA3E,HECW1,SRRM4,FSTL4,GFRA2,NEC-TIN1,NGFR,KDR,CNTN2,SHANK1,NTRK3,GRB10,GRIN1,LZTS1,EPHA4
GO:0048545	response to steroid hormone	3.86E-03	26	582	CDKN1A,ACVR1,S100B,CCL2,CYP7B1,VEGFA,WNT5A,AQP4,TXNIP,IGFBP2,PRSS8,SREBF1,DAB2,BCL3,PTGDS,GPR83,SPHK1,CACNA1G,MGARP,ABHD2,NTRK3,NR1H3,CXCL1,LMNB1,EIF4EBP1,TNF
GO:0003013	circulatory system process	3.86E-03	33	838	SLC24A3,KCNIP1,FABP5,CACNG3,SEMA3A,ADRA1A,HEG1,KCNK6,CXCL12,VEGFA,SLC13A3,MYZAP,CCM2L,P2RX2,AOC3,OCLN,ATP2A3,ATP2B2,CXCL10,SREB

				F1,BCR,KCNJ2,ABCC8,GLP1R,CACNA1G,AG-TRAP,CACNA1C,NPPC,RGS2,SLC7A3,P2RY1,TNF,EPAS1
GO:0040017	positive regulation of locomotion	4.51E-03	30	734	ACVR1,PGF,SEMA3A,RIN2,CCL2,GPNMB,MMP14,CXCL12,VEGFA,ONE-CUT2,SEMA4B,WNT5A,CXCL14,FAM83H,MAPRE1,AOC3,SEMA3E,HDAC9,MYLK,CXCL10,DAB2,SMOC2,NFE2L2,SPHK1,KDR,NTRK3,GRIN1,LGALS3,TNF,CDH13
GO:0010975	regulation of neuron projection development	4.64E-03	27	627	CHN1,SEMA3A,CXCL12,CREB3L2,VEGFA,SEMA4B,WNT5A,PPFIA2,CRMP1,STYXL1,SEMA3E,HECW1,DAB2,FSTL4,DGKG,GFAP,NFE2L2,SPHK1,NGFR,CNTN2,NEU4,NTRK3,GRIN1,RGS2,LZTS1,LTK,EPHA4
GO:0034330	cell junction organization	5.63E-03	34	899	LRTM2,WNT4,HEG1,PKP1,FGFR2,MMP14,VEGFA,WNT5A,PPFIA2,SLITRK3,P2RX2,SLI-TRK5,OCLN,ATP2B2,SEMA3E,GABRB2,MYLK,THSD1,BCR,LIN7B,ABCC8,NECTIN1,KDR,MAR-VELD3,CNTN2,SHANK1,NTRK3,GRIN1,GRM5,TNF,RASSF8,CDH13,PHLDB2,EPHA4
GO:0001763	morphogenesis of a branching structure	5.63E-03	16	270	LGR4,WNT4,ACVR1,PGF,SEMA3A,FGFR2,MMP14,CXCL12,VEGFA,WNT5A,CELSR1,SEMA3E,MYCN,KDR,TNF,CSMD1
GO:1902284	neuron projection extension involved in neuron projection guidance	5.92E-03	7	51	WNT4,SEMA3A,CXCL12,VEGFA,SEMA4B,WNT5A,SEMA3E
GO:0048846	axon extension involved in axon guidance	5.92E-03	7	51	WNT4,SEMA3A,CXCL12,VEGFA,SEMA4B,WNT5A,SEMA3E
GO:0030516	regulation of axon extension	6.60E-03	11	138	WNT4,SEMA3A,CXCL12,VEGFA,SEMA4B,WNT5A,SEMA3E,FSTL4,CNTN2,NTRK3,EPHA4
GO:0050927	positive regulation of positive chemotaxis	6.69E-03	6	36	PGF,CXCL12,VEGFA,KDR,NTRK3,CDH13
GO:0009611	response to wounding	7.13E-03	34	919	CDKN1A,WNT4,FABP5,TYRO3,SCN3A,CCL2,FGFR2,CXCL12,CREB3L2,VEGFA,WNT5A,CELSR1,CCM2L,P2RX2,PRKAR2A,OCLN,MYLK,DGKG,SMOC2,GFAP,NFE2L2,ABCC8,NGFR,KDR,CNTN2,ABHD2,DOCK9,NTRK3,CXCL1,P2RX3,P2RY1,TNF,PHLDB2,EPHA4
GO:0050926	regulation of positive chemotaxis	7.21E-03	6	37	PGF,CXCL12,VEGFA,KDR,NTRK3,CDH13
GO:0048675	axon extension	7.21E-03	13	194	WNT4,SEMA3A,CXCL12,VEGFA,COL6A3,SEMA4B,WNT5A,AUTS2,SEMA3E,FSTL4,CNTN2,NTRK3,EPHA4
GO:0007409	axonogenesis	7.41E-03	29	732	RPS6KA5,LGR4,LRTM2,WNT4,CHN1,SEMA3A,S100B,FGFR2,MMP14,CXCL12,VEGFA,COL6A3,SEMA4B,WNT5A,SLITRK3,CRMP1,SLI-TRK5,AUTS2,SEMA3E,SRRM4,FSTL4,GFRA2,NEC-TIN1,NGFR,CNTN2,NTRK3,GRB10,GRIN1,EPHA4
GO:0008016	regulation of heart contraction	7.69E-03	17	315	KCNIP1,CACNG3,SEMA3A,ADRA1A,KCNK6,VEGFA,ATP2A3,ATP2B2,SREBF1,KCNJ2,GLP1R,CACNA1G,CACNA1C,NPPC,RGS2,TNF,EPAS1
GO:0034329	cell junction assembly	7.83E-03	23	517	LRTM2,WNT4,HEG1,PKP1,MMP14,VEGFA,WNT5A,SLITRK3,SLI-TRK5,OCLN,GABRB2,MYLK,THSD1,BCR,LIN7B,NEC-TIN1,KDR,MAR-VELD3,SHANK1,NTRK3,GRIN1,TNF,PHLDB2

GO:0042391	regulation of membrane potential	7.95E-03	24	554	KCNIP1,CACNG3,ADRA1A,SCN3A,PIP5KL1,KCNK6,KCNK13,P2RX2,GABRB2,KCNB1,KCNC1,KCNJ2,NGFR,KCNQ3,KDR,CACNA1G,SHANK1,CACNA1C,NTRK3,GRIN1,IGSF11,GRM5,P2RX3,TNF
GO:1901653	cellular response to peptide	7.98E-03	24	556	ENPP1,TLR6,AP3S1,CCL2,ATP6V1C2,PRKAR2A,PNPLA3,HDAC9,SREBF1,NFE2L2,KLF5,GLP1R,NGFR,MGARP,AGTRAP,CACNA1C,RAB31,GRB10,NR1H3,GRIN1,GRM5,CCND2,EIF4EBP1,EPHA4
GO:0046660	female sex differentiation	7.98E-03	13	199	WNT4,FZD4,TYRO3,MMP14,MMP19,VEGFA,WNT5A,ACVR1C,DACH2,KDR,CAPN5,CCND2,CSMD1
GO:0042471	ear morphogenesis	9.02E-03	13	202	GRXCR1,MYO3B,FGFR2,PLS1,WNT5A,CELSR1,FRZB,ATP2B2,BCR,MAFB,DLX3,GSC,ADGRG6
GO:0031344	regulation of cell projection organization	1.03E-02	33	911	CHN1,FZD4,SEMA3A,MYO3B,PLS1,CXCL12,CREB3L2,VEGFA,SEMA4B,WNT5A,PPFIA2,CRMP1,AUTS2,STYXL1,OCLN,SEMA3E,HECW1,DAB2,FSTL4,DGKG,GFAP,NFE2L2,SPHK1,KLF5,NGFR,CNTN2,NEU4,NTRK3,GRIN1,RGS2,LZTS1,LTK,EPHA4
GO:0007411	axon guidance	1.03E-02	20	427	RPS6KA5,LGR4,LRTM2,WNT4,CHN1,SEMA3A,MMP14,CXCL12,VEGFA,COL6A3,SEMA4B,WNT5A,CRMP1,SEMA3E,GFRA2,NECTIN1,NGFR,CNTN2,GRB10,EPHA4
GO:0097485	neuron projection guidance	1.03E-02	20	428	RPS6KA5,LGR4,LRTM2,WNT4,CHN1,SEMA3A,MMP14,CXCL12,VEGFA,COL6A3,SEMA4B,WNT5A,CRMP1,SEMA3E,GFRA2,NECTIN1,NGFR,CNTN2,GRB10,EPHA4
GO:1901888	regulation of cell junction assembly	1.03E-02	14	236	LRTM2,WNT4,MMP14,VEGFA,WNT5A,SLITRK3,SLITRK5,OCLN,NECTIN1,KDR,NTRK3,GRIN1,TNF,PHLDB2
GO:1903959	regulation of anion transmembrane transport	1.03E-02	11	152	ENPP1,FABP5,AZIN1,AOC3,OCLN,NFE2L2,GLP1R,GRB10,GRM5,RGS2,TNF
GO:0042472	inner ear morphogenesis	1.12E-02	12	181	GRXCR1,MYO3B,FGFR2,PLS1,WNT5A,CELSR1,FRZB,ATP2B2,BCR,MAFB,DLX3,ADGRG6
GO:0048511	rhythmic process	1.12E-02	20	433	LGR4,FZD4,TYRO3,CYP7B1,MMP19,ENOX2,MAGEL2,WNT5A,BHLHE40,AZIN1,NMS,SREBF1,PTGDS,GDF10,NGFR,ZPBP2,CAPN5,NTRK3,NR1H3,GRIN1
GO:0090596	sensory organ morphogenesis	1.26E-02	23	544	GRXCR1,MYO3B,FGFR2,MMP14,PLS1,VEGFA,SFRP5,WNT5A,AQP5,CELSR1,FRZB,ATP2B2,MYLK,BCL6,BCR,PROM1,NECTIN1,KDR,MAFB,DLX3,GSC,ADGRG6,EPHA4
GO:0090066	regulation of anatomical structure size	1.30E-02	26	657	WNT4,SEMA3A,ADRA1A,MYO3B,PLS1,CXCL12,VEGFA,SEMA4B,MAGEL2,WNT5A,AQP4,SLC12A7,ATP2B2,SEMA3E,FSTL4,CACNA1G,CNTN2,CACNA1C,NPPC,NTRK3,RGS2,ARPC1B,P2RY1,TNF,HCK,EPHA4
GO:0120035	regulation of plasma membrane bounded cell projection organization	1.30E-02	32	891	CHN1,SEMA3A,MYO3B,PLS1,CXCL12,CREB3L2,VEGFA,SEMA4B,WNT5A,PPFIA2,CRMP1,AUTS2,STYXL1,OCLN,SEMA3E,HECW1,DAB2,FSTL4,DGKG,GFAP,NFE2L2,SPHK1,KLF5,NGFR,CNTN2,NEU4,NTRK3,GRIN1,RGS2,LZTS1,LTK,EPHA4
GO:0033198	response to ATP	1.44E-02	6	45	CCL2,CIB2,P2RX2,SPHK1,P2RX3,P2RY1
GO:0061387	regulation of extent of cell growth	1.47E-02	11	161	WNT4,SEMA3A,CXCL12,VEGFA,SEMA4B,WNT5A,SEMA3E,FSTL4,CNTN2,NTRK3,EPHA4
GO:0050768	negative regulation of neurogenesis	1.47E-02	12	189	SEMA3A,SEMA4B,WNT5A,SEMA3E,MYCN,FSTL4,BRINP1,ABCC8,NGFR,NTRK3,TNF,EPHA4

GO:0060047	heart contraction	1.48E-02	19	412	KCNIP1,CACNG3,SEMA3A,ADRA1A,KCNK6,VEGFA,MYZAP,CCM2L,ATP2A3,ATP2B2,SREBF1,KCNJ2,GLP1R,CACNA1G,CACNA1C,NPPC,RGS2,TNF,EPAS1
GO:0031960	response to corticosteroid	1.48E-02	17	345	CDKN1A,ACVR1,S100B,CCL2,VEGFA,WNT5A,AQP4,IGFBP2,PRSS8,PTGDS,GPR83,CACNA1G,NTRK3,CXCL1,LMNB1,EIF4EBP1,TNF
GO:0043506	regulation of JUN kinase activity	1.53E-02	9	111	TLR6,FZD4,MAP3K9,SFRP5,WNT5A,MAPKBP1,DAB2,TNF,EPHA4
GO:0051384	response to glucocorticoid	1.53E-02	16	314	CDKN1A,ACVR1,S100B,CCL2,VEGFA,WNT5A,AQP4,IGFBP2,PTGDS,GPR83,CACNA1G,NTRK3,CXCL1,LMNB1,EIF4EBP1,TNF
GO:0050770	regulation of axonogenesis	1.61E-02	13	222	CHN1,SEMA3A,CXCL12,VEGFA,SEMA4B,WNT5A,SEMA3E,FSTL4,NGFR,CNTN2,NTRK3,GRIN1,EPHA4
GO:0016053	organic acid biosynthetic process	1.66E-02	18	385	SDSL,FABP5,ACSL3,CYP7B1,PLOD2,MTHFR,GAD2,ACSM1,SREBF1,PTGDS,ELOVL2,GGT1,OSBPL2,HPGDS,SPHK1,ABHD2,NR1H3,LDHA
GO:0001952	regulation of cell-matrix adhesion	1.66E-02	10	139	WNT4,RIN2,MMP14,VEGFA,ONECUT2,SEMA3E,BCL6,KDR,CDH13,PHLDB2
GO:0008015	blood circulation	1.66E-02	28	755	KCNIP1,CACNG3,SEMA3A,ADRA1A,KCNK6,CXCL12,VEGFA,MYZAP,CCM2L,P2RX2,AOC3,OCLN,ATP2A3,ATP2B2,CXCL10,SREBF1,BCR,KCNJ2,ABCC8,GLP1R,CACNA1G,AGTRAP,CACNA1C,NPPC,RGS2,P2RY1,TNF,EPAS1
GO:0051961	negative regulation of nervous system development	1.66E-02	12	195	SEMA3A,SEMA4B,WNT5A,SEMA3E,MYCN,FSTL4,BRINP1,ABCC8,NGFR,NTRK3,TNF,EPHA4
GO:0003015	heart process	1.66E-02	19	421	KCNIP1,CACNG3,SEMA3A,ADRA1A,KCNK6,VEGFA,MYZAP,CCM2L,ATP2A3,ATP2B2,SREBF1,KCNJ2,GLP1R,CACNA1G,CACNA1C,NPPC,RGS2,TNF,EPAS1
GO:0043410	positive regulation of MAPK cascade	1.66E-02	25	641	TLR6,FZD4,SEMA3A,ADRA1A,MAP3K9,CCL2,FGFR2,GPNMB,PRXL2C,CXCL12,VEGFA,WNT5A,MAPKBP1,RASSF2,DAB2,SPHK1,NGFR,KDR,NTRK3,GRM5,TFF2,LMNB1,P2RY1,TNF,EPHA4
GO:1990138	neuron projection extension	1.66E-02	14	256	WNT4,RNF157,SEMA3A,CXCL12,VEGFA,COL6A3,SEMA4B,WNT5A,AUTS2,SEMA3E,FSTL4,CNTN2,NTRK3,EPHA4
GO:0061564	axon development	1.66E-02	29	797	RPS6KA5,LGR4,LRTM2,WNT4,CHN1,SEMA3A,S100B,FGFR2,MMP14,CXCL12,VEGFA,COL6A3,SEMA4B,WNT5A,SLITRK3,CRMP1,SLITRK5,AUTS2,SEMA3E,SRRM4,FSTL4,GFRA2,NECTIN1,NGFR,CNTN2,NTRK3,GRB10,GRIN1,EPHA4
GO:0008361	regulation of cell size	1.68E-02	14	257	WNT4,SEMA3A,CXCL12,VEGFA,SEMA4B,WNT5A,AQP4,SLC12A7,ATP2B2,SEMA3E,FSTL4,CNTN2,NTRK3,EPHA4
GO:0043507	positive regulation of JUN kinase activity	1.69E-02	8	91	TLR6,FZD4,MAP3K9,WNT5A,MAPKBP1,DAB2,TNF,EPHA4
GO:0071300	cellular response to retinoic acid	1.80E-02	8	92	FZD4,CCL2,FGFR2,WNT5A,BRINP3,BRINP1,NTRK3,LTK
GO:0030308	negative regulation of cell growth	1.91E-02	13	231	CDKN1A,ENPP1,SEMA3A,SEMA4B,WNT5A,FRZB,VGLL4,SEMA3E,DAB2,FSTL4,BCL6,RGS2,EPHA4
GO:0046683	response to organophosphorus	1.91E-02	12	201	ENPP1,CCL2,CYP7B1,MMP19,CIB2,P2RX2,SREBF1,SPHK1,LDHA,CCND2,P2RX3,P2RY1
GO:0098656	anion transmembrane transport	1.91E-02	24	614	ENPP1,SLC24A3,FABP5,MFSD4A,SLC13A3,SLC7A14,AZIN1,AOC3,SLC17A8,OCLN,SLC12A7,ANO3,GABRB2,BCR,GFAP,NFE2L2,GLP1R,SLC25A21,GRB10,GRM5,RGS2,SLC7A3,SLC16A3,TNF

GO:0007160	cell-matrix adhesion	1.91E-02	14	263	WNT4,RIN2,MMP14,VEGFA,ONECUT2,PPFIA2,SEMA3E,BCL6,THSD1,BCR,KDR,CNTN2,CDH13,PHLDB2
GO:0044057	regulation of system process	1.91E-02	29	810	KCNIP1,FABP5,CACNG3,SEMA3A,S100B,ADRA1A,KCNK6,VEGFA,ATP2A3,ATP2B2,SREBF1,DAB2,SPHK1,KCNJ2,ABCC8,GLP1R,NGFR,CACNA1G,SHANK1,CACNA1C,NPPC,NR1H3,GRIN1,IGSF11,RGS2,TFF2,P2RX3,TNF,EPAS1
GO:0099177	regulation of trans-synaptic signaling	1.91E-02	26	692	FABP5,CACNG3,S100B,ADRA1A,CCL2,PPFIA2,BHLHE40,P2RX2,PRKAR2A,ATP2B2,BCR,GFAP,KCNB1,SPHK1,NGFR,KDR,CNTN2,SHANK1,GRIN1,IGSF11,GRM5,P2RX3,P2RY1,LZTS1,TNF,EPHA4
GO:0045596	negative regulation of cell differentiation	1.91E-02	31	891	CRIM1,ENPP1,WNT4,SEMA3A,S100B,VEGFA,SEMA4B,WNT5A,CXCL14,FRZB,SEMA3E,MYCN,CXCL10,FSTL4,BRINP1,BCL6,GDF10,NFE2L2,KLF5,ABCC8,NGFR,MAFB,CNTN2,NPPC,NTRK3,NR1H3,PRAMEF12,RGS2,S1PR3,TNF,EPHA4
GO:0046545	development of primary female sexual characteristics	1.98E-02	11	174	WNT4,FZD4,MMP14,MMP19,VEGFA,ACVR1C,DACH2,KDR,CAPN5,CCND2,CSMD1
GO:0019932	second-messenger-mediated signaling	1.99E-02	26	696	LGR4,RNF157,ADRA1A,CCL2,VEGFA,MT1A,P2RX2,PRKAR2A,CXCL10,SPHK1,GLP1R,KDR,CACNA1C,NPPC,GPR17,GRIN1,GRM5,RGS2,S1PR3,TFF2,ADGRG6,P2RX3,P2RY1,TNF,RXFP1,CDH13
GO:1905603	regulation of blood-brain barrier permeability	1.99E-02	3	8	VEGFA,OCLN,ABCC8
GO:0002687	positive regulation of leukocyte migration	1.99E-02	11	175	PGF,CCL2,MMP14,CXCL12,VEGFA,WNT5A,CXCL14,AOC3,CXCL10,LGALS3,TNF
GO:0048640	negative regulation of developmental growth	1.99E-02	10	148	CDKN1A,SEMA3A,SEMA4B,WNT5A,IGFBP2,VGLL4,SEMA3E,FSTL4,RGS2,EPHA4
GO:0001525	angiogenesis	1.99E-02	30	859	ACVR1,PGF,SEMA3A,CCL2,FGFR2,GPNMB,MMP14,MMP19,CXCL12,VEGFA,WNT5A,IGFBP2,SEMA3E,HDAC9,CXCL10,DAB2,SMOC2,NFE2L2,SPHK1,KLF5,ABCC8,NGFR,KDR,RALB,CXCL1,LGALS3,ADGRG6,TNF,EPAS1,CDH13
GO:0003018	vascular process in circulatory system	1.99E-02	16	334	SLC24A3,FABP5,ADRA1A,VEGFA,SLC13A3,OCLN,ATP2A3,BCR,ABCC8,CACNA1G,CACNA1C,NPPC,RGS2,SLC7A3,P2RY1,TNF
GO:0050921	positive regulation of chemotaxis	1.99E-02	11	177	PGF,CCL2,CXCL12,VEGFA,WNT5A,CXCL14,CXCL10,SMOC2,KDR,NTRK3,CDH13
GO:1900721	positive regulation of uterine smooth muscle relaxation	1.99E-02	2	2	ABCC8,NPPC
GO:1900719	regulation of uterine smooth muscle relaxation	1.99E-02	2	2	ABCC8,NPPC
GO:1901551	negative regulation of endothelial cell development	1.99E-02	2	2	VEGFA,S1PR3
GO:0044558	uterine smooth muscle relaxation	1.99E-02	2	2	ABCC8,NPPC
GO:1903141	negative regulation of	1.99E-02	2	2	VEGFA,S1PR3

	establishment of endothelial barrier				
GO:0048638	regulation of developmental growth	2.03E-02	20	476	CDKN1A,WNT4,RNF157,SEMA3A,FGFR2,PLS1,CXCL12,VEGFA,SEMA4B,WNT5A,IGFBP2,VGLL4,SEMA3E,FSTL4,CNTN2,CACNA1C,NPPC,NTRK3,RGS2,EPHA4
GO:0051960	regulation of nervous system development	2.04E-02	24	626	LRTM2,SEMA3A,S100B,CXCL12,VEGFA,SEMA4B,WNT5A,SLITRK3,BHLHE40,SLITRK5,SEMA3E,MYCN,RNF112,FSTL4,BRINP1,GFAP,ABCC8,NGFR,KDR,CNTN2,NTRK3,GRM5,TNF,EPHA4
GO:0035637	multicellular organismal signaling	2.16E-02	13	240	KCNIP1,CACNG3,SCN3A,KCNK6,P2RX2,ATP2A3,ATP2B2,KCNJ2,CACNA1G,CACNA1C,NPPC,NTRK3,P2RX3
GO:0035590	purinergic nucleotide receptor signaling pathway	2.32E-02	5	36	P2RX2,P2RY13,CNTN2,P2RX3,P2RY1
GO:1901652	response to peptide	2.32E-02	28	792	ENPP1,TLR6,AP3S1,CCL2,ATP6V1C2,CXCL12,ACVR1C,PRKAR2A,PNPLA3,HDAC9,PRSS8,SREBF1,NFE2L2,KLF5,ABCC8,GLP1R,NGFR,MGARP,AGTRAP,CACNA1C,RAB31,GRB10,NR1H3,GRIN1,GRM5,CCND2,EIF4EBP1,EPHA4
GO:0001508	action potential	2.32E-02	12	212	KCNIP1,CACNG3,ADRA1A,SCN3A,P2RX2,KCNB1,KCNJ2,CACNA1G,CACNA1C,NTRK3,P2RX3,TNF
GO:0030517	negative regulation of axon extension	2.32E-02	6	55	SEMA3A,SEMA4B,WNT5A,SEMA3E,FSTL4,EPHA4
GO:0050919	negative chemotaxis	2.32E-02	6	55	LGR4,LRTM2,SEMA3A,SEMA4B,WNT5A,SEMA3E
GO:0046394	carboxylic acid biosynthetic process	2.34E-02	17	377	FABP5,ACSL3,CYP7B1,PLOD2,MTHFR,GAD2,ACSM1,SREBF1,PTGDS,ELOVL2,GGT1,OSBPL2,HPGDS,SPHK1,ABHD2,NR1H3,LDHA
GO:0030324	lung development	2.41E-02	14	277	HEG1,FGFR2,MMP14,VEGFA,WNT5A,SIM2,CELSR1,MYCN,SREBF1,KDR,EIF4EBP1,TNF,RXFP1,EPAS1
GO:0006874	cellular calcium ion homeostasis	2.41E-02	22	561	SLC24A3,S100B,ADRA1A,CCL2,CXCL12,CIB2,WNT5A,P2RX2,ATP2A3,ATP2B2,CXCL10,GLP1R,KDR,CACNA1G,CACNA1C,GPR17,GRIN1,GRM5,CXCL1,S1PR3,P2RX3,P2RY1
GO:0090036	regulation of protein kinase C signaling	2.47E-02	4	21	ADRA1A,VEGFA,WNT5A,DGKG
GO:0072503	cellular divalent inorganic cation homeostasis	2.47E-02	23	601	SLC24A3,S100B,ADRA1A,CCL2,CXCL12,CIB2,WNT5A,MT1A,P2RX2,ATP2A3,ATP2B2,CXCL10,GLP1R,KDR,CACNA1G,CACNA1C,GPR17,GRIN1,GRM5,CXCL1,S1PR3,P2RX3,P2RY1
GO:0045926	negative regulation of growth	2.57E-02	15	313	CDKN1A,ENPP1,SEMA3A,SEMA4B,WNT5A,MT1A,IGFBP2,FRZB,VGLL4,SEMA3E,DAB2,FSTL4,BCL6,RGS2,EPHA4
GO:0050920	regulation of chemotaxis	2.57E-02	14	280	PGF,SEMA3A,CCL2,CXCL12,VEGFA,SEMA4B,WNT5A,CXCL14,SEMA3E,CXCL10,SMOC2,KDR,NTRK3,CDH13
GO:0031589	cell-substrate adhesion	2.57E-02	18	418	WNT4,FZD4,TYRO3,RIN2,MMP14,VEGFA,ONECUT2,PPFIA2,SEMA3E,DAB2,BCL6,THSD1,SMOC2,BCR,KDR,CNTN2,CDH13,PHLDB2
GO:1904646	cellular response to amyloid-beta	2.57E-02	6	57	TLR6,NGFR,CACNA1C,GRIN1,GRM5,EPHA4
GO:0043408	regulation of MAPK cascade	2.57E-02	30	884	TLR6,FZD4,SEMA3A,ADRA1A,MAP3K9,CCL2,FGFR2,GPNMB,PRXL2C,CXCL12,VEGFA,SFRP5,WNT5A,CNK

					SR3,MAP-KBP1,RASSF2,DAB2,SPHK1,NGFR,KDR,MAR-VELD3,NTRK3,PRAMEF12,GRM5,RGS2,TFF2,LMNB1,P2RY1,TNF,EPHA4
GO:0030323	respiratory tube development	2.57E-02	14	281	HEG1,FGFR2,MMP14,VEGFA,WNT5A,SIM2,CELSR1,MYCN,SREBF1,KDR,EIF4EBP1,TNF,RXFP1,EPAS1
GO:0048588	developmental cell growth	2.67E-02	16	350	WNT4,RNF157,SEMA3A,ADRA1A,CXCL12,VEGFA,COL6A3,SEMA4B,WNT5A,AUTS2,SEMA3E,FSTL4,CNTN2,NTRK3,RGS2,EPHA4
GO:0051593	response to folic acid	2.75E-02	4	22	MGMT,MOG,VEGFA,MTHFR
GO:0060562	epithelial tube morphogenesis	2.75E-02	22	572	LGR4,WNT4,ACVR1,PGF,SEMA3A,FGFR2,MMP14,CXCL12,VEGFA,SFRP5,WNT5A,CELSR1,MTHFR,SEMA3E,MYCN,CXCL10,KDR,DLX3,CACNA1C,TNF,CSMD1,EPHA4
GO:0071677	positive regulation of mononuclear cell migration	2.75E-02	7	81	CCL2,CXCL12,WNT5A,CXCL14,CXCL10,LGALS3,TNF
GO:0034764	positive regulation of transmembrane transport	2.75E-02	14	285	CACNG3,CCL2,AZIN1,CNKSR3,AOC3,OCLN,PRSS8,CXCL10,KCNC1,NFE2L2,KCNJ2,ABCC8,GLP1R,CACNA1C
GO:0034767	positive regulation of ion transmem-brane transport	2.75E-02	14	285	CACNG3,CCL2,AZIN1,CNKSR3,AOC3,OCLN,PRSS8,CXCL10,KCNC1,NFE2L2,KCNJ2,ABCC8,GLP1R,CACNA1C
GO:0060541	respiratory system development	2.75E-02	15	319	HEG1,FGFR2,MMP14,VEGFA,WNT5A,SIM2,CELSR1,MYCN,SREBF1,KDR,DLX3,EIF4EBP1,TNF,RXFP1,EPAS1
GO:0002053	positive regulation of mesenchymal cell proliferation	2.75E-02	5	39	FGFR2,VEGFA,WNT5A,MYCN,KDR
GO:0050804	modulation of chemical synaptic transmission	2.75E-02	25	691	CACNG3,S100B,ADRA1A,CCL2,PPFIA2,BHLHE40,P2RX2,PRKAR2A,ATP2B2,BCR,GFAP,KCNB1,SPHK1,NGFR,KDR,CNTN2,SHANK1,GRIN1,IGSF11,GRM5,P2RX3,P2RY1,LZTS1,TNF,EPHA4
GO:0001667	ameboidal-type cell migration	2.75E-02	25	691	WNT4,ACVR1,LTB4R2,SEMA3A,HMCN2,RIN2,MMP14,CXCL12,VEGFA,SEMA4B,WNT5A,ACVR1C,CELSR1,SH3PXD2A,SEMA3E,HDAC9,SMOC2,NFE2L2,KDR,MARVELD3,IGSF11,LPP,TNF,LTK,CDH13
GO:0010810	regulation of cell-substrate adhesion	2.75E-02	13	253	WNT4,FZD4,RIN2,MMP14,VEGFA,ONE-CUT2,SEMA3E,DAB2,BCL6,SMOC2,KDR,CDH13,PHLDB2
GO:0001569	branching in-volved in blood vessel morpho-genesis	2.75E-02	6	59	ACVR1,SEMA3A,CXCL12,VEGFA,SEMA3E,KDR
GO:0033552	response to vita-min B3	2.75E-02	3	10	FABP5,CCL2,PMP2
GO:0010977	negative regula-tion of neuron projection devel-opment	2.75E-02	11	191	SEMA3A,SEMA4B,WNT5A,CRMP1,SEMA3E,DAB2,FSTL4,GFAP,NGFR,NEU4,EPHA4
GO:0061042	vascular wound healing	2.94E-02	4	23	VEGFA,SMOC2,KDR,TNF
GO:0060284	regulation of cell development	2.99E-02	25	697	SEMA3A,CXCL12,VEGFA,SEMA4B,WNT5A,BHLHE40,FRZB,SEMA3E,MYCN,HDAC9,RNF112,DAB2,FSTL4,BRINP1,GFAP,ABCC8,NGFR,KDR,CNTN2,NPPC,NTRK3,GRM5,S1PR3,TNF,EPHA4

GO:1903522	regulation of blood circulation	3.02E-02	17	394	KCNIP1,CACNG3,SEMA3A,ADRA1A,KCNK6,VEGFA,ATP2A3,ATP2B2,SREBF1,KCNJ2,GLP1R,CACNA1G,CACNA1C,NPPC,RGS2,TNF,EPAS1
GO:0055074	calcium ion homeostasis	3.09E-02	22	582	SLC24A3,S100B,ADRA1A,CCL2,CXCL12,CIB2,WNT5A,P2RX2,ATP2A3,ATP2B2,CXCL10,GLP1R,KDR,CACNA1G,CACNA1C,GPR17,GRIN1,GRM5,CXCL1,S1PR3,P2RX3,P2RY1
GO:0050771	negative regulation of axonogenesis	3.09E-02	7	84	SEMA3A,SEMA4B,WNT5A,SEMA3E,FSTL4,NGFR,EPHA4
GO:0032570	response to progesterone	3.09E-02	7	84	CCL2,VEGFA,TXNIP,SREBF1,SPHK1,ABHD2,NR1H3
GO:0051928	positive regulation of calcium ion transport	3.09E-02	10	165	CCL2,CXCL12,P2RX2,ATP2B2,MYLK,CXCL10,CACNA1C,GRIN1,LGALS3,P2RX3
GO:1903034	regulation of response to wounding	3.17E-02	12	227	WNT4,CCL2,CXCL12,OCLN,MYLK,SMOC2,NFE2L2,ABCC8,NTRK3,TNF,PHLDB2,EPHA4
GO:0071318	cellular response to ATP	3.27E-02	4	24	CCL2,CIB2,P2RX3,P2RY1
GO:0032787	monocarboxylic acid metabolic process	3.29E-02	26	745	PDK1,PFKL,FABP5,ACSL3,CKM,CYP7B1,PRXL2C,PNPLA3,CYB5A,GAD2,AUH,ACSM1,SREBF1,PTGDS,ELOVL2,GGT1,OSBPL2,HPGDS,SPHK1,SLC27A3,ABHD2,NR1H3,LDHA,SLC7A3,SLC16A3,ENO2
GO:0010827	regulation of glucose transmembrane transport	3.31E-02	8	111	ENPP1,FABP5,AOC3,OCLN,NFE2L2,GLP1R,GRB10,TNF
GO:0090037	positive regulation of protein kinase C signaling	3.38E-02	3	11	ADRA1A,VEGFA,WNT5A
GO:0008585	female gonad development	3.39E-02	10	168	WNT4,FZD4,MMP14,MMP19,VEGFA,ACVR1C,KDR,CAPN5,CCND2,CSMD1
GO:0071805	potassium ion transmembrane transport	3.40E-02	12	230	SLC24A3,KCNIP1,KCNK6,KCNT1,KCNK13,SLC12A7,KCNB1,KCNC1,KCNJ2,ABCC8,KCNQ3,KCNG3
GO:0072330	monocarboxylic acid biosynthetic process	3.43E-02	13	263	FABP5,ACSL3,CYP7B1,ACSM1,SREBF1,PTGDS,ELOVL2,OSBPL2,HPGDS,SPHK1,ABHD2,NR1H3,LDHA
GO:0031279	regulation of cyclase activity	3.46E-02	14	297	LGR4,LTB4R2,ADRA1A,CXCL10,P2RY13,GLP1R,CACNA1C,RCVRN,RGS2,S1PR3,ADGRG6,P2RY1,RXFP1,RD3
GO:0072507	divalent inorganic cation homeostasis	3.55E-02	23	632	SLC24A3,S100B,ADRA1A,CCL2,CXCL12,CIB2,WNT5A,MT1A,P2RX2,ATP2A3,ATP2B2,CXCL10,GLP1R,KDR,CACNA1G,CACNA1C,GPR17,GRIN1,GRM5,CXCL1,S1PR3,P2RX3,P2RY1
GO:0001558	regulation of cell growth	3.55E-02	21	555	CDKN1A,ENPP1,WNT4,RNF157,SEMA3A,MMP14,CXCL12,VEGFA,SEMA4B,WNT5A,FRZB,VGLL4,SEMA3E,DAB2,FSTL4,BCL6,SPHK1,CNTN2,NTRK3,RGS2,EPHA4
GO:1905604	negative regulation of blood-brain barrier permeability	3.55E-02	2	3	VEGFA,ABCC8
GO:1901082	positive regulation of relaxation of smooth muscle	3.55E-02	2	3	ABCC8,NPPC
GO:0032349	positive regulation of aldosterone biosynthetic process	3.55E-02	2	3	WNT4,DAB2

GO:0032346	positive regulation of aldosterone metabolic process	3.55E-02	2	3	WNT4,DAB2
GO:0014074	response to purine-containing compound	3.55E-02	12	233	ENPP1,CCL2,CYP7B1,MMP19,CIB2,P2RX2,SREBF1,SPHK1,LDHA,CCND2,P2RX3,P2RY1
GO:0046777	protein autophosphorylation	3.55E-02	13	266	ENPP1,TYRO3,MYO3B,MAP3K9,FGFR2,GPNMB,VEGFA,RASSF2,BCR,GFRA2,KDR,HCK,EPHA4
GO:0042074	cell migration involved in gastrulation	3.55E-02	7	88	WNT4,ACVR1,MMP14,CXCL12,WNT5A,CELSR1,LPP
GO:0050767	regulation of neurogenesis	3.55E-02	20	518	SEMA3A,CXCL12,VEGFA,SEMA4B,WNT5A,BHLHE40,SEMA3E,MYCN,RNF112,FSTL4,BRINP1,GFAP,ABCC8,NGFR,KDR,CNTN2,NTRK3,GRM5,TNF,EPHA4
GO:0040008	regulation of growth	3.55E-02	29	879	CDKN1A,ENPP1,WNT4,RNF157,SEMA3A,FGFR2,MMP14,PLS1,CXCL12,VEGFA,ENOX2,SEMA4B,WNT5A,MT1A,IGFBP2,FRZB,VGLL4,SEMA3E,DAB2,FSTL4,BCL6,SPHK1,CNTN2,CACNA1C,NPPC,NTRK3,IGSF11,RGS2,EPHA4
GO:0051339	regulation of lyase activity	3.62E-02	14	301	LGR4,LTB4R2,ADRA1A,CXCL10,P2RY13,GLP1R,CACNA1C,RCVRN,RGS2,S1PR3,ADGRG6,P2RY1,RXFP1,RD3
GO:0032535	regulation of cellular component size	3.62E-02	19	482	WNT4,SEMA3A,MYO3B,PLS1,CXCL12,VEGFA,SEMA4B,MAGEL2,WNT5A,AQP4,SLC12A7,ATP2B2,SEMA3E,FSTL4,CNTN2,NTRK3,ARPC1B,HCK,EPHA4
GO:0048562	embryonic organ morphogenesis	3.62E-02	23	637	CRIM1,GRXCR1,WNT4,ACVR1,MYO3B,FGFR2,MMP14,PLS1,SFRP5,WNT5A,CELSR1,FRZB,ATP2B2,MYCN,BCR,MAFB,DLX3,CACNA1C,NR1H3,GSC,ADGRG6,EPHA4,DCAF7
GO:0060688	regulation of morphogenesis of a branching structure	3.62E-02	6	65	LGR4,PGF,FGFR2,VEGFA,WNT5A,TNF
GO:0032963	collagen metabolic process	3.63E-02	9	143	WNT4,CCL2,MMP10,MMP14,MMP19,PLOD2,SERPINB7,NPPC,P3H2
GO:0044283	small molecule biosynthetic process	3.66E-02	27	800	SDSL,ENPP1,WNT4,FABP5,ACSL3,CYP7B1,PLOD2,AMPD3,MTHFR,GAD2,ACSM1,SREBF1,DAB2,PTGDS,ELOVL2,GGT1,OSBPL2,HPGDS,SPHK1,ABHD2,NTHL1,NR1H3,LDHA,P2RY1,TNF,PCBD1,ENO2
GO:0042060	wound healing	3.86E-02	24	681	CDKN1A,WNT4,TYRO3,CCL2,FGFR2,VEGFA,WNT5A,CELSR1,CCM2L,P2RX2,PRKAR2A,OCLN,MYLK,DGKG,SMOC2,NFE2L2,ABCC8,KDR,DOCK9,CXCL1,P2RX3,P2RY1,TNF,PHLDB2
GO:0035249	synaptic transmission, glutamatergic	3.94E-02	8	117	CACNG3,CCL2,SLC17A8,NGFR,SHANK1,GRIN1,GRM5,TNF
GO:0009612	response to mechanical stimulus	3.94E-02	16	376	ENPP1,OTOF,CCL2,MMP14,CXCL12,CIB2,TXNIP,IGFBP2,ATP2B2,ANO3,CXCL10,KCNC1,KCNJ2,P2RX3,P2RY1,TNF
GO:0090075	relaxation of muscle	3.99E-02	5	45	KCNJ2,ABCC8,NPPC,RGS2,P2RY1
GO:0040013	negative regulation of locomotion	4.02E-02	18	451	WNT4,SEMA3A,PIP5KL1,CCL2,CXCL12,SEMA4B,WNT5A,ACVR1C,SEMA3E,BCR,NFE2L2,ABCC8,NGFR,MARVELD3,ABHD2,GRM5,TNF,PHLDB2
GO:0016049	cell growth	4.02E-02	24	685	CDKN1A,ENPP1,WNT4,RNF157,SEMA3A,ADRA1A,MMP14,CXCL12,VEGFA,COL6A3,SEMA4B,WNT5A,FRZB,AUTS2,VGLL4,SEMA3E,DAB2,FSTL4,BCL6,SPHK1,CNTN2,NTRK3,RGS2,EPHA4

GO:0032870	cellular response to hormone stimulus	4.02E-02	29	891	ENPP1,LGR4,ACVR1,PGF,AP3S1,CCL2,CYP7B1,ATP6V1C2,PLOD2,VEGFA,IGFBP2,PRKAR2A,PNPLA3,HDAC9,SREBF1,DAB2,NFE2L2,GLP1R,CACNA1G,MGARP,AGTRAP,RAB31,ABHD2,GRB10,NR1H3,CCND2,LMNB1,EIF4EBP1,RXFP1
GO:0070098	chemokine-mediated signaling pathway	4.14E-02	7	92	CCL2,CXCL12,ACKR1,CXCL10,GPR17,CXCL1,TFF2
GO:1904659	glucose transmembrane transport	4.14E-02	9	147	ENPP1,FABP5,MFSD4A,AOC3,OCLN,NFE2L2,GLP1R,GRB10,TNF
GO:0006665	sphingolipid metabolic process	4.29E-02	10	178	GBA3,B3GALT2,SERINC2,ELOVL2,SPHK1,ZPBP2,NEU4,SPTSSB,TNF,LARGE1
GO:0032526	response to retinoic acid	4.29E-02	10	178	FZD4,CCL2,FGFR2,WNT5A,IGFBP2,BRINP3,SREBF1,BRINP1,NTRK3,LTK
GO:0007610	behavior	4.37E-02	30	940	GCNT4,FZD4,S100B,CYP7B1,CXCL12,MTMR12,CIB2,BHLHE40,CELSR1,P2RX2,AOC3,SLITRK5,AUTS2,ATP2B2,NMS,BRINP1,PTGDS,HPGDS,ABCC8,GLP1R,CNTN2,SHANK1,CACNA1C,GRIN1,GRM5,CCND2,P2RX3,P2RY1,CSMD1,EPHA4
GO:0060191	regulation of lipase activity	4.42E-02	11	211	ADRA1A,FGFR2,VEGFA,ARL1,HDAC9,GPR17,NTRK3,NR1H3,GRM5,RGS2,P2RY1
GO:0009313	oligosaccharide catabolic process	4.42E-02	3	13	MANBA,GBA3,NEU4
GO:0014832	urinary bladder smooth muscle contraction	4.42E-02	3	13	P2RX2,CACNA1C,P2RX3
GO:0060601	lateral sprouting from an epithelium	4.42E-02	3	13	FGFR2,WNT5A,CELSR1
GO:0019371	cyclooxygenase pathway	4.42E-02	3	13	PTGDS,HPGDS,SPHK1
GO:0048041	focal adhesion assembly	4.42E-02	7	94	WNT4,MMP14,VEGFA,THSD1,BCR,KDR,PHLDB2
GO:0010464	regulation of mesenchymal cell proliferation	4.42E-02	5	47	FGFR2,VEGFA,WNT5A,MYCN,KDR
GO:1903960	negative regulation of anion transmembrane transport	4.42E-02	5	47	ENPP1,FABP5,GRB10,RGS2,TNF
GO:0048843	negative regulation of axon extension involved in axon guidance	4.42E-02	4	28	SEMA3A,SEMA4B,WNT5A,SEMA3E
GO:0008645	hexose transmembrane transport	4.42E-02	9	150	ENPP1,FABP5,MFSD4A,AOC3,OCLN,NFE2L2,GLP1R,GRB10,TNF
GO:0048880	sensory system development	4.48E-02	29	903	FABP5,FZD4,SEMA3A,FGFR2,MMP14,CXCL12,VEGFA,PMP2,CIB2,SFRP5,WNT5A,AQP5,SLC7A14,SH3PXD2A,SLC17A8,ATP2B2,MYLK,BCL6,PROM1,NECTIN1,NGFR,KDR,DLX3,CACNA1C,NTRK3,NR1H3,ADGRG6,EPHA4,RD3
GO:0015749	monosaccharide transmembrane transport	4.79E-02	9	152	ENPP1,FABP5,MFSD4A,AOC3,OCLN,NFE2L2,GLP1R,GRB10,TNF
GO:0048247	lymphocyte chemotaxis	4.86E-02	6	71	CCL2,CYP7B1,CXCL12,WNT5A,CXCL14,CXCL10

GO:1904645	response to amyloid-beta	4.86E-02	6	71	TLR6,NGFR,CACNA1C,GRIN1,GRM5,EPHA4
GO:0061061	muscle structure development	4.87E-02	31	995	CDKN1A,WNT4,ACVR1,S100B,ADRA1A,HEG1,FGFR2,MMP14,PLOD2,CXCL12,HSPB2,VEGFA,COL6A3,MTMR12,WNT5A,CXCL14,CCM2L,P2RX2,HDAC9,MYLK,VAMP5,CXCL10,DAB2,KLF5,NGFR,TAGLN,GSC,RGS2,TNF,EPAS1,LARGE1
GO:0048569	post-embryonic animal organ development	4.87E-02	4	29	GRXCR1,VEGFA,KDR,LDHA
GO:2000241	regulation of reproductive process	4.96E-02	12	249	ZFP57,WNT4,SEMA3A,VEGFA,WNT5A,ACVR1C,PRKAR2A,PTGDS,HPGDS,NPPC,CLXN,P2RY1
GO:0031667	response to nutrient levels	4.97E-02	25	744	CDKN1A,WNT4,FABP5,ACSL3,MGMT,CCL2,MOG,VEGFA,PMP2,ACVR1C,IGFBP2,MTHFR,PNPLA3,CXCL10,SREBF1,KCNB1,NFE2L2,SPHK1,RALB,NR1H3,LDHA,CXCL1,TBL1XR1,KPTN,EIF4EBP1
GO:0060560	developmental growth involved in morphogenesis	5.00E-02	16	391	WNT4,RNF157,SEMA3A,FGFR2,CXCL12,VEGFA,COL6A3,SEMA4B,SFRP5,WNT5A,AUTS2,SEMA3E,FSTL4,CNTN2,NTRK3,EPHA4

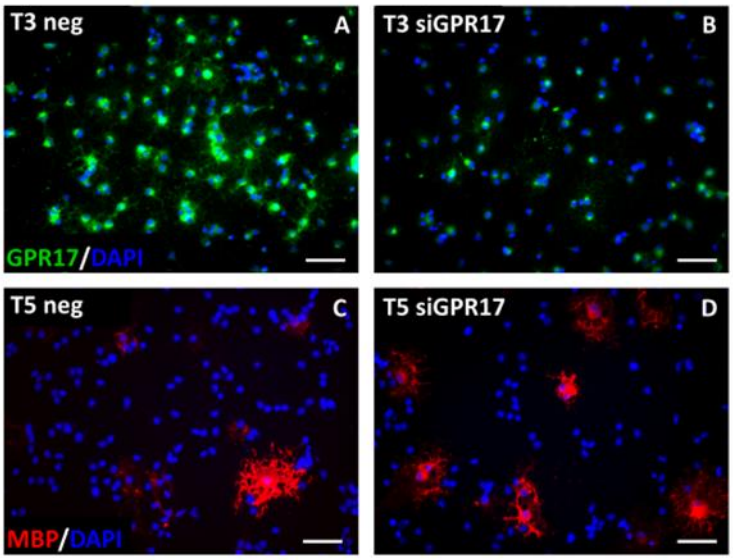

Figure S2. Effect of GPR17 silencing on OPC maturation. Representative pictures at 20X magnification showing GPR17- (in green at T3) and MBP- (in red at T5) positive cells in control OPC cultures (A, C) and after GPR17 silencing (B, D). In blue, staining of cell nuclei. Scale bar: 50μm.

Table S3. Descriptive tables of metabolomic analysis. The tables show the fold changes (and the relative p-values) of all metabolites, calculated comparing their abundance in GPR17-silenced versus control OPCs at DID 1 (A), DID 2 (B), DID 3 (C), DID 5 (D). Fisher's LSD corrected by FDR; p.adj<0.1.

**A) Metabolic profile siGPR17 vs neg - DID 1**				
**Metabolite**	**Fold change (FC)**	**log2(FC)**	**p.adjusted**	**-log10(p)**
Arg	1.5669	0.64787	0.24121	0.61761
Lys	1.5184	0.60257	0.24121	0.61761
C14-Carnitine	0.71517	-0.48363	0.24121	0.61761
Ac-Ornithine	1.5126	0.59706	0.39615	0.40214
Ornithine	1.397	0.48229	0.39615	0.40214
Gln	1.3139	0.39382	0.39615	0.40214
Asp	1.1185	0.16156	0.39615	0.40214
His	1.5922	0.67106	0.48651	0.31291
3-phosphoglycerate/2-phosphoglycerate	1.3396	0.42175	0.48651	0.31291
C16-Carnitine	0.76188	-0.39237	0.48651	0.31291
Citrate	1.3036	0.38255	0.48651	0.31291
Glucose	1.2668	0.34121	0.48651	0.31291
Glu	1.1932	0.25486	0.48651	0.31291
C5-Carnitine	0.85511	-0.22581	0.48651	0.31291
Fumarate	0.87218	-0.1973	0.48651	0.31291
Sedoheptulose phosphate	0.89787	-0.15542	0.48651	0.31291
C181-Carnitine	0.68481	-0.54622	0.48989	0.3099
Tyr	1.2852	0.36197	0.48989	0.3099
Noraepinephrine	1.2672	0.34162	0.48989	0.3099
Thr	1.2602	0.3336	0.48989	0.3099
Val	1.2361	0.30577	0.48989	0.3099
Trp	1.2269	0.295	0.48989	0.3099
Ser	1.1863	0.24642	0.48989	0.3099
Alpha Keto glutarate	0.88437	-0.17727	0.48989	0.3099
Asn	1.3242	0.40517	0.49705	0.3036
Phosphoenolpyruvate	1.4067	0.49229	0.50118	0.30001
Met	1.4038	0.48937	0.50118	0.30001
Phe	1.2802	0.35636	0.50118	0.30001
6-phosphoglycerate	1.1617	0.21629	0.50118	0.30001
Sarcosine	1.1607	0.21503	0.50118	0.30001
Ile	1.1557	0.2088	0.50118	0.30001
Gly	1.1424	0.19211	0.50118	0.30001
Glucose-6P	1.0711	0.099107	0.50118	0.30001
Leu	1.1658	0.22133	0.59992	0.22191
DHAP/GAP	1.1357	0.18361	0.59992	0.22191
C3-Carnitine	0.91217	-0.13262	0.59992	0.22191
C180-Carnitine	1.1344	0.18195	0.60313	0.21959
Creatine	1.1497	0.20125	0.68598	0.16369
Pro	1.1227	0.16696	0.68598	0.16369
Met-SO	1.1057	0.14491	0.68598	0.16369
Ala	1.1033	0.14186	0.68598	0.16369
Succinate	1.0885	0.12232	0.68598	0.16369
Carnosine	1.0774	0.1076	0.68598	0.16369

Malate	0.95966	-0.059401	0.68598	0.16369
C10-Carnitine	1.3893	0.47431	0.68766	0.16262
Acetyl coA	0.92512	-0.11228	0.68766	0.16262
Lactate	0.94969	-0.074465	0.68766	0.16262
Erythrose 4-Phosphate	1.0468	0.065976	0.68766	0.16262
Free Carnitine	0.96315	-0.054166	0.68766	0.16262
Ribulose 5P/R5P	1.0332	0.047173	0.68766	0.16262
AMP	0.96845	-0.046249	0.68766	0.16262
C2-Carnitine	1.0356	0.050455	0.75479	0.12217
Taurine	1.1171	0.15973	0.77585	0.11022
C4-Carnitine	0.98123	-0.027334	0.88398	0.053557

**B) Metabolic profile siGPR17 vs neg - DID 2**				
**Metabolite**	**Fold change (FC)**	**log2(FC)**	**p.adjusted**	**-log10(p)**
Glucose	0.64094	-0.64174	0.000075926	4.1196
AMP	1.3256	0.40664	0.00030108	3.5213
Malate	1.2491	0.32088	0.00039241	3.4063
Lactate	0.56559	-0.82217	0.00098907	3.0048
Fumarate	1.2502	0.32211	0.001834	2.7366
C4-Carnitine	1.5389	0.62193	0.0042958	2.367
Creatine	0.7045	-0.50533	0.0088659	2.0523
C3-Carnitine	1.5965	0.6749	0.011109	1.9543
Ornithine	0.7989	-0.32391	0.015443	1.8113
C2-Carnitine	1.3273	0.40849	0.020606	1.686
Ribulose 5P/R5P	1.4967	0.5818	0.021488	1.6678
C5-Carnitine	1.4304	0.51639	0.040135	1.3965
Free Carnitine	1.4356	0.52168	0.043675	1.3598
Citrate	1.2361	0.30574	0.04464	1.3503
C16-Carnitine	1.7101	0.77406	0.078489	1.1052
Met	1.3457	0.42834	0.12613	0.89917
Ala	1.3396	0.42181	0.16137	0.79219
Noraepinephrine	0.86348	-0.21176	0.17189	0.76474
C181-Carnitine	2.0696	1.0494	0.18965	0.72205
Carnosine	0.8605	-0.21675	0.23186	0.63478
3-phosphoglycerate/2-phosphoglycerate	1.1392	0.18804	0.2527	0.59739
C14-Carnitine	0.78119	-0.35626	0.28056	0.55197
Sarcosine	1.1932	0.25481	0.29241	0.53402
Asp	1.384	0.46882	0.30863	0.51057
Alpha Keto glutarate	0.94671	-0.079007	0.31431	0.50264
Glu	1.3893	0.47441	0.31721	0.49865
Gly	1.223	0.29041	0.32989	0.48163
Glucose 6P	0.9208	-0.11904	0.33024	0.48118
Thr	1.1614	0.21583	0.35812	0.44597
Succinate	1.1386	0.18726	0.35832	0.44573
Trp	1.1291	0.17515	0.35865	0.44533
Pro	1.1721	0.22904	0.40805	0.38929
C180-Carnitine	1.4078	0.49348	0.41651	0.38038
Sedoheptulose phosphate	1.1388	0.18754	0.41784	0.37899
Met-SO	0.88756	-0.17209	0.44566	0.35099

Asn	1.0663	0.09265	0.45868	0.33849
Phosphoenolpyruvate	1.1728	0.22998	0.48121	0.31767
Ile	1.0641	0.089691	0.48939	0.31034
Tyr	1.0778	0.1081	0.53206	0.27404
Taurine	1.1181	0.16101	0.54403	0.26437
Ser	1.0685	0.09563	0.56625	0.24699
Phe	1.0671	0.093663	0.59044	0.22883
Gln	0.93736	-0.093324	0.60493	0.2183
Ac-Ornithine	1.1035	0.14208	0.61208	0.21319
Val	1.0541	0.076001	0.66033	0.18024
Arg	1.0799	0.11083	0.74665	0.12688
Acetyl coA	1.0179	0.025591	0.77203	0.11236
Leu	1.0237	0.033738	0.78916	0.10283
Erythrose 4-Phosphate	1.0169	0.024193	0.81143	0.090747
6-phosphoglycerate	1.0199	0.028392	0.90672	0.042526
Lys	0.97602	-0.035013	0.91684	0.037707
His	0.99172	-0.012001	0.95543	0.019801
DHAP/GAP	0.99424	-0.0083384	0.95721	0.018994
C10-Carnitine	1.0151	0.021579	0.97311	0.011839

C) Metabolic profile siGPR17 vs neg - DID 3				
**Metabolite**	**Fold change (FC)**	**log2(FC)**	**p.adjusted**	**-log10(p)**
Lactate	0.30202	-1.7273	8.6478E-07	6.0631
Noraepinephrine	0.54447	-0.87707	0.0020744	2.6831
Carnosine	0.54482	-0.87615	0.0061389	2.2119
C3-Carnitine	1.769	0.8229	0.077599	1.1101
Creatine	0.56479	-0.82422	0.084301	1.0742
Met	2.1241	1.0869	0.1435	0.84315
Phosphoenolpyruvate	2.4505	1.2931	0.17292	0.76215
Asp	1.5658	0.64693	0.17292	0.76215
Glu	1.4158	0.50164	0.17292	0.76215
3-phosphoglycerate/2-phosphoglycerate	3.0213	1.5952	0.2346	0.62967
Ac-Ornithine	1.8585	0.89416	0.2346	0.62967
His	1.8531	0.88997	0.2346	0.62967
Free Carnitine	1.6586	0.72997	0.2346	0.62967
Arg	1.6546	0.72649	0.2346	0.62967
Fumarate	1.6324	0.70695	0.2346	0.62967
Pro	1.6322	0.70678	0.2346	0.62967
Gly	1.5508	0.63302	0.2346	0.62967
Phe	1.5095	0.59405	0.2346	0.62967
Ser	1.5054	0.59013	0.2346	0.62967
Trp	1.4962	0.58132	0.2346	0.62967
Tyr	1.4749	0.56057	0.2346	0.62967
Thr	1.4691	0.55496	0.2346	0.62967
C14-Carnitine	0.6807	-0.55491	0.2346	0.62967
Gln	1.4064	0.49201	0.2346	0.62967
C2-Carnitine	1.4039	0.48946	0.2346	0.62967
Val	1.3913	0.47643	0.2346	0.62967
Succinate	0.73163	-0.45081	0.2346	0.62967

Sarcosine	1.3627	0.44651	0.2346	0.62967
C4-Carnitine	1.3516	0.43462	0.2346	0.62967
Malate	1.2917	0.3693	0.2346	0.62967
Ala	1.2741	0.34943	0.2346	0.62967
Lys	1.4681	0.55398	0.27777	0.55632
Ile	1.4573	0.54329	0.27777	0.55632
Asn	1.3919	0.47708	0.27777	0.55632
C5-Carnitine	1.2543	0.32691	0.27777	0.55632
Leu	1.2575	0.33054	0.2809	0.55145
Ribulose 5P/R5P	0.88577	-0.175	0.28434	0.54616
Citrate	1.367	0.45096	0.28475	0.54554
DHAP/GAP	0.83511	-0.25997	0.35457	0.4503
Glucose	0.82891	-0.2707	0.38629	0.41309
C16-Carnitine	1.1907	0.25176	0.42233	0.37435
Sedoheptulose phosphate	1.1902	0.25119	0.52125	0.28295
Acetyl-coA	1.6098	0.68689	0.5365	0.27043
Met-SO	0.85863	-0.21989	0.54374	0.26461
C181-Carnitine	1.1148	0.15673	0.58493	0.23289
Alpha Keto glutarate	1.1203	0.16389	0.66018	0.18034
Glucose-6P	0.93438	-0.097912	0.72733	0.13827
AMP	0.91331	-0.13082	0.80393	0.09478
C10-Carnitine	1.1439	0.19395	0.88426	0.053422
Taurine	1.1397	0.18867	0.88426	0.053422
6-phosphoglycerate	1.0818	0.11349	0.88426	0.053422
Erythrose 4-Phosphate	0.9662	-0.049613	0.88426	0.053422
C180-Carnitine	0.96008	-0.058777	0.89718	0.04712
Ornithine	1.0296	0.042145	0.89718	0.04712

**D) Metabolic profile siGPR17 vs neg - DID 5**				
**Metabolite**	**Fold change (FC)**	**log2(FC)**	**p.adjusted**	**-log10(p)**
Met-SO	0.45225	-1.1448	2.5192E-10	9.5987
Free Carnitine	1.6447	0.71781	0.000044502	4.3516
C3-Carnitine	1.4767	0.5624	0.00013288	3.8765
C4-Carnitine	1.5469	0.62934	0.00033004	3.4814
C181-Carnitine	2.2592	1.1758	0.00033294	3.4776
Taurine	0.56738	-0.81761	0.00040936	3.3879
C14-Carnitine	1.7283	0.78939	0.00062787	3.2021
C180-Carnitine	1.5452	0.62782	0.0018217	2.7395
Creatine	0.60387	-0.7277	0.0045399	2.343
3-phosphoglycerate/2-phosphoglycerate	1.784	0.83512	0.016678	1.7778
Carnosine	0.7412	-0.43206	0.025107	1.6002
C2-Carnitine	1.262	0.33566	0.025501	1.5934
Acetyl-coA	0.69708	-0.5206	0.027999	1.5529
Citrate	1.4048	0.49036	0.035297	1.4523
DHAP/GAP	1.8446	0.88333	0.047336	1.3248
C5-Carnitine	1.1876	0.24807	0.055822	1.2532
Fumarate	1.8763	0.90793	0.0782	1.1068
Ile	0.86118	-0.21561	0.082933	1.0813
C16-Carnitine	3.196	1.6762	0.088794	1.0516
Ornithine	0.80713	-0.30913	0.11669	0.93296

Asp	1.2755	0.35112	0.13326	0.87529
Noraepinephrine	0.81145	-0.30142	0.14607	0.83543
Met	1.2787	0.35468	0.15318	0.8148
Trp	0.88279	-0.17986	0.19033	0.72048
Malate	1.2743	0.34975	0.19952	0.70002
Val	0.91916	-0.12162	0.20851	0.68087
Ribulose 5P/R5P	1.3858	0.47073	0.20932	0.67918
Asn	0.90194	-0.14889	0.23775	0.62388
Phosphoenolpyruvate	1.4252	0.51116	0.23994	0.61989
Leu	0.88474	-0.17668	0.26953	0.56939
Thr	0.89292	-0.16339	0.27457	0.56135
Lactate	0.83428	-0.2614	0.31359	0.50363
6-phosphoglycerate	1.4613	0.54726	0.3507	0.45506
His	0.9075	-0.14004	0.44938	0.34739
Sedoheptulose phosphate	0.92262	-0.11619	0.4719	0.32615
Phe	0.94186	-0.08641	0.55649	0.25454
Tyr	0.94255	-0.085361	0.61702	0.2097
AMP	1.1468	0.19761	0.62971	0.20086
Glu	0.94322	-0.084337	0.64199	0.19247
Erythrose 4-Phosphate	0.94781	-0.077324	0.65863	0.18136
Ser	1.0311	0.044185	0.70354	0.15271
Alpha Keto glutarate	1.0409	0.057837	0.71053	0.14842
Pro	0.97123	-0.042108	0.71559	0.14534
Succinate	0.94126	-0.087336	0.71803	0.14386
C10-Carnitine	1.156	0.20913	0.73442	0.13405
Arg	1.0452	0.063766	0.77756	0.10927
Gly	0.98642	-0.019733	0.82943	0.081218
Glucose-6P	1.032	0.045511	0.83073	0.080539
Glucose	0.98248	-0.025495	0.8878	0.051686
Sarcosine	1.0125	0.017868	0.91269	0.039675
Gln	1.0134	0.019171	0.92054	0.035957
Ac-Ornithine	1.0068	0.0097292	0.96918	0.013596
Ala	0.99729	-0.003918	0.98031	0.0086355
Lys	1.0014	0.0020421	0.99309	0.0030118

Figure S3. Effect of AR-C155858 on lactate release. Lactate abundance in the extracellular space (medium) was measured after treatment of GPR17-silenced (siGPR) and control (neg) OPCs with AR-C155858 (MCT1 IN). Data are presented as mean ± SD from 2 independent experiments.

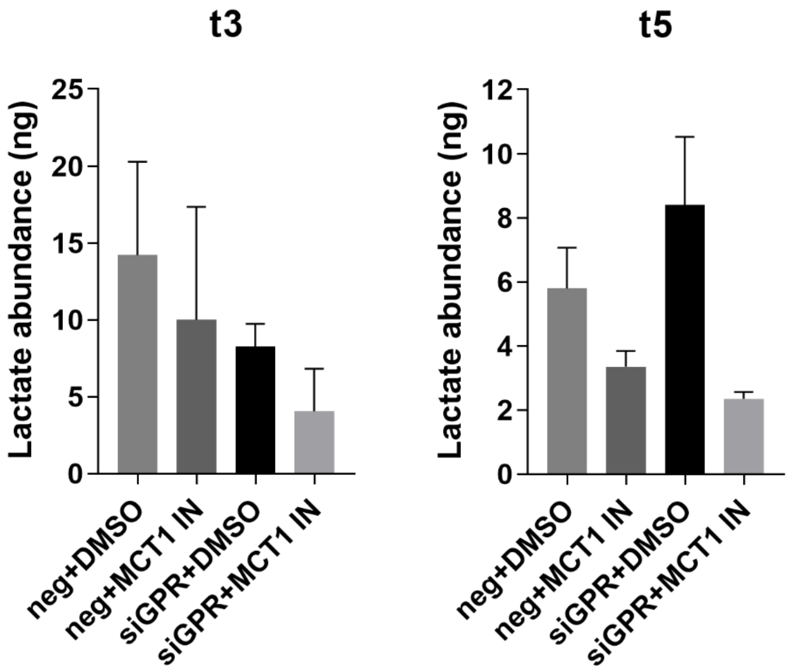

Table S4. Descriptive tables of lipidomic analysis. The tables show the fold changes (and the relative p-values) for all lipids, calculated comparing their abundance in GPR17-silenced versus control OPCs at DID 3 (A), DID 5 (B). Fisher's LSD corrected by FDR; p.adj<0.1). PE= phosphatidylethanolamine; PC= phosphatidylcholine; aa= diacyl; ae= acyl-alkyl; SM = sphingomyelin; Cer= ceramide; GCer= Glycolsil-ceramide.

A) Lipidomic profile siGPR17 vs neg - DID 3				
**Lipid**	**Fold change (FC)**	**log2(FC)**	**p.ajusted**	**-LOG10(p)**
PE aa 34:3	1.9353	0.95256	0.042192	1.3748
PC aa C40:4	0.6871	-0.5414	0.076325	1.1173
PE aa 32:1	2.5302	1.3393	0.11498	0.9394
PE aa 32:2	2.2171	1.1487	0.11498	0.9394
SM C18:0	1.8389	0.87887	0.11498	0.9394
lysoPC a C24:0	1.6548	0.72662	0.11498	0.9394
lysoPC a C20:3	1.6544	0.72629	0.11498	0.9394
PC aa C40:5	0.61587	-0.69929	0.11498	0.9394
PC aa C42:5	0.61917	-0.69158	0.11498	0.9394
PC aa C38:4	0.70173	-0.51102	0.11498	0.9394
PE aa 40:6	0.71881	-0.47633	0.11498	0.9394
lysoPC a C16:1	2.2121	1.1454	0.11679	0.93261
PE ae 36:1	1.9432	0.95843	0.11679	0.93261
PC aa C34:2	1.5971	0.67548	0.11679	0.93261
PC ae C36:2	1.5466	0.62909	0.11679	0.93261
PE aa 36:3	1.5363	0.6195	0.11679	0.93261
PE ae 38:4	0.75718	-0.40129	0.11679	0.93261
SM (OH) C22:1	1.5003	0.58527	0.11901	0.92443
PE aa 42:5	0.60445	-0.72631	0.13668	0.86431
PC ae C30:0	1.6529	0.72501	0.13668	0.86431
lysoPC a C18:2	2.0628	1.0446	0.14659	0.8339
PC ae C42:0	0.66185	-0.59542	0.14659	0.8339
PC aa C42:4	0.66298	-0.59296	0.14659	0.8339
PC aa C32:2	1.7997	0.84773	0.15092	0.82124
lysoPC a C14:0	2.3788	1.2503	0.15784	0.80178
PC aa C38:5	0.62678	-0.67397	0.15784	0.80178
PC aa C36:4	0.62728	-0.67282	0.15784	0.80178
PC aa C34:3	1.5729	0.65343	0.15784	0.80178
SM (OH) C16:1	1.5544	0.63638	0.15784	0.80178
PC ae C36:1	1.4557	0.54169	0.15784	0.80178
PC aa C40:3	0.80283	-0.31683	0.15784	0.80178
PC ae C44:3	2.2011	1.1382	0.15844	0.80014
PC ae C44:4	2.3793	1.2506	0.16631	0.77909
SM C18:1	1.5054	0.5901	0.16631	0.77909
PC aa C32:1	1.4444	0.53048	0.16631	0.77909
PE aa 40:4	0.71702	-0.47991	0.17674	0.75266
PE aa 34:4	2.288	1.1941	0.1783	0.74885
PC ae C42:1	0.55538	-0.84846	0.1783	0.74885
PE aa 36:5	1.6178	0.69406	0.1783	0.74885
PC aa C40:6	0.6252	-0.67761	0.1783	0.74885
PE aa 40:5	0.68707	-0.54147	0.1783	0.74885
PE ae 42:0	0.74884	-0.41727	0.1783	0.74885
SM (OH) C24:1	0.61739	-0.69576	0.19474	0.71053
PC ae C38:2	1.3991	0.48452	0.21194	0.67379
lysoPE a C20:4	1.9296	0.94833	0.21584	0.66587

lysoPC a C28:1	1.3809	0.46565	0.21584	0.66587
PE aa 36:8	1.7566	0.8128	0.22263	0.65241
GCer-d18:1/18:0	1.6635	0.73424	0.22263	0.65241
PC aa C42:6	0.62995	-0.66669	0.22263	0.65241
PC ae C32:1	1.5566	0.63843	0.22263	0.65241
PC ae C30:1	1.6404	0.71409	0.23692	0.62541
lysoPE a C18:0	1.8921	0.91997	0.24003	0.61973
PC aa C32:0	1.3675	0.45155	0.25146	0.59952
PE ae 34:0	1.486	0.57141	0.25473	0.59391
SM C16:0	1.4188	0.50464	0.25473	0.59391
PE aa 36:6	1.8516	0.8888	0.27083	0.5673
PC aa C36:3	1.2657	0.33995	0.27256	0.56453
PE ae 38:0	1.606	0.6835	0.27425	0.56185
PE aa 38:6	1.3515	0.43461	0.27425	0.56185
PE aa 34:1	1.394	0.47919	0.27845	0.55525
PC ae C38:5	0.52448	-0.93104	0.29364	0.53218
PS C38:3	0.55054	-0.86107	0.29364	0.53218
PI C38:3	0.56616	-0.82072	0.29364	0.53218
PS C38:4	1.7243	0.78599	0.29364	0.53218
PE aa 38:7	1.4984	0.58343	0.29364	0.53218
PC ae C32:2	1.4959	0.58097	0.29364	0.53218
lysoPC a C17:0	1.3547	0.438	0.29364	0.53218
PE aa 38:8	1.2578	0.33088	0.29364	0.53218
PE aa 34:2	1.527	0.61069	0.29598	0.52874
PE aa 42:4	0.67576	-0.56543	0.29598	0.52874
PE aa 40:3	0.70032	-0.51392	0.29598	0.52874
PE ae 40:4	0.7379	-0.43851	0.32136	0.493
lysoPC a C16:0	1.2527	0.32506	0.32136	0.493
PE aa 44:6	0.66698	-0.58428	0.32538	0.48761
PE aa 42:9	0.69103	-0.53318	0.33801	0.47106
PE ae 38:6	1.2854	0.36218	0.33801	0.47106
PC aa C42:2	0.72935	-0.45532	0.3385	0.47044
PE ae 40:5	0.79733	-0.32675	0.35748	0.44675
PE aa 42:8	0.76056	-0.39487	0.3578	0.44636
PE ae 38:5	1.3001	0.37867	0.3578	0.44636
PC aa C36:6	1.2948	0.37269	0.3578	0.44636
PE aa 32:0	2.0952	1.0671	0.36009	0.44359
PE aa 34:0	1.3148	0.3948	0.37241	0.42898
GCer-d18:1/16:0	1.5565	0.63828	0.37257	0.42879
PC aa C30:0	1.3054	0.38449	0.37257	0.42879
PE ae 40:1	1.5836	0.66323	0.37384	0.42731
PC ae C42:2	0.66233	-0.59437	0.37384	0.42731
PI C38:4	0.70787	-0.49844	0.37384	0.42731
PC aa C30:2	1.304	0.3829	0.37384	0.42731
lysoPE a C16:0	1.5228	0.60668	0.3758	0.42505
lysoPC a C26:0	1.4018	0.48725	0.3758	0.42505
PE aa 42:6	0.77213	-0.37308	0.3758	0.42505
PE aa 40:9	1.2861	0.36298	0.3758	0.42505
PC ae C34:0	1.1942	0.25607	0.37583	0.42501
PC aa C32:3	1.3244	0.40538	0.37913	0.42121
PC ae C40:5	0.6488	-0.62416	0.38212	0.4178
Linoleic acid	1.2962	0.37427	0.38212	0.4178
PC aa C34:4	1.2201	0.28702	0.38212	0.4178

PE ae 38:2	1.2708	0.34571	0.40078	0.3971
PC ae C34:2	1.2882	0.36537	0.40314	0.39454
PE aa 36:4	1.4249	0.51083	0.4369	0.35962
Cer-d18:1/24:1	0.70624	-0.50177	0.44037	0.35619
PE aa 36:2	0.80128	-0.31963	0.44496	0.35168
PC ae C40:6	0.62817	-0.67078	0.44509	0.35155
SM (OH) C14:1	1.4759	0.56158	0.46166	0.33568
PC ae C38:1	1.2859	0.36277	0.46214	0.33522
SM C20:2	1.2129	0.27847	0.47316	0.32499
PC ae C38:6	0.66195	-0.5952	0.47712	0.32137
PC ae C38:0	1.1493	0.20081	0.47712	0.32137
PC aa C34:1	1.1056	0.14482	0.47712	0.32137
Arachidonic acid	0.70357	-0.50723	0.48082	0.31802
PS C40:7	1.37	0.45417	0.52104	0.28313
lysoPC a C18:1	1.1841	0.24375	0.52104	0.28313
PS C40:5	1.314	0.39392	0.52711	0.2781
PC ae C44:6	1.3102	0.38973	0.52711	0.2781
PC ae C40:4	0.80703	-0.30931	0.5307	0.27515
PC aa C38:0	0.55219	-0.85675	0.53551	0.27124
PC ae C40:1	0.75643	-0.40273	0.53551	0.27124
PC ae C30:2	1.2626	0.33635	0.53551	0.27124
lysoPC a C26:1	1.1803	0.2392	0.55639	0.25462
PC ae C38:4	0.80196	-0.3184	0.57442	0.24077
PE aa 40:8	1.1526	0.20491	0.57442	0.24077
PE ae 42:1	0.71541	-0.48317	0.59335	0.22669
lysoPE a C18:1	1.3425	0.42491	0.59335	0.22669
PS C36:2	1.258	0.33115	0.59335	0.22669
PC ae C36:4	0.80536	-0.31229	0.59335	0.22669
PC aa C28:1	1.2131	0.27871	0.59335	0.22669
PS C38:5	1.178	0.23634	0.59335	0.22669
PE aa 38:0	1.1464	0.19706	0.59335	0.22669
PC aa C36:2	1.1092	0.14954	0.59335	0.22669
lysoPE a C14:0	0.48468	-1.0449	0.5978	0.22344
lysoPE a C18:2	1.6094	0.68651	0.5978	0.22344
Palmitic acid	0.74211	-0.4303	0.5978	0.22344
PE ae 40:3	0.75701	-0.40161	0.5978	0.22344
lysoPC a C18:0	0.77393	-0.36973	0.5978	0.22344
PC ae C40:3	1.171	0.22771	0.5978	0.22344
PC aa C40:2	0.89107	-0.16638	0.5978	0.22344
PC aa C38:3	0.92631	-0.11043	0.5978	0.22344
lysoPC a C20:4	0.803	-0.31652	0.61839	0.20874
PC ae C34:1	1.095	0.13092	0.63543	0.19693
PE aa 38:2	1.1461	0.19678	0.63586	0.19664
SM C24:1	1.0719	0.1002	0.63586	0.19664
PE ae 38:1	0.78158	-0.35553	0.63804	0.19515
PC aa C26:0	1.1411	0.19045	0.64147	0.19283
PC ae C36:3	1.1234	0.16788	0.64362	0.19137
Stearic acid	0.77738	-0.3633	0.66701	0.17587
PC aa C24:0	1.1068	0.14636	0.66932	0.17437
PC ae C44:5	1.2883	0.36547	0.68441	0.16468
PC ae C40:2	1.1556	0.20859	0.71434	0.1461
PI C38:5	0.87425	-0.19389	0.7243	0.14008
Cer-d18:1/18:0	1.08	0.11103	0.7243	0.14008

PC ae C38:3	1.0745	0.1036	0.7243	0.14008
PC ae C36:5	0.68993	-0.53548	0.73054	0.13636
PE aa 40:2	0.92608	-0.11078	0.74069	0.13036
PS C40:6	1.0435	0.061459	0.7585	0.12004
PC ae C34:3	1.0501	0.070501	0.76945	0.11382
PE aa 40:10	0.93712	-0.093699	0.77364	0.11146
PE aa 40:7	1.0476	0.067151	0.77364	0.11146
PE ae 38:3	1.0762	0.10589	0.78592	0.10462
PE aa 38:3	1.0672	0.093888	0.78592	0.10462
SM C26:1	0.95089	-0.072643	0.78592	0.10462
lysoPC a C28:0	1.035	0.049654	0.78592	0.10462
Oleic acid	1.0282	0.040066	0.78592	0.10462
PE ae 40:0	1.0339	0.048108	0.80092	0.09641
PE aa 42:10	0.90273	-0.14764	0.81092	0.091022
Cer-d18:1/16:0	0.81294	-0.29878	0.83163	0.08007
PE aa 44:9	0.91588	-0.12677	0.84395	0.073685
PC aa C36:1	0.92255	-0.1163	0.85622	0.067417
SM C24:0	1.0587	0.082279	0.86551	0.062729
PC aa C38:1	0.91586	-0.1268	0.86551	0.062729
PE aa 38:5	0.94118	-0.087461	0.87154	0.059711
GCer-d18:1/24:1	0.9056	-0.14305	0.88325	0.053915
Margaric acid	0.85034	-0.23389	0.88845	0.051365
SM C26:0	1.0172	0.024612	0.89767	0.046886
PE aa 42:7	0.91827	-0.12301	0.89767	0.046886
Nervonic acid	0.91211	-0.13272	0.89767	0.046886
PC aa C36:5	0.88699	-0.17301	0.89767	0.046886
SM C22:3	0.88699	-0.17301	0.89767	0.046886
PE aa 38:1	1.0141	0.020245	0.90636	0.042701
PE aa 38:4	0.96676	-0.048764	0.90636	0.042701
SM C16:1	0.9299	-0.10486	0.90636	0.042701
PE aa 40:1	0.8765	-0.19017	0.90636	0.042701
PE ae 40:7	0.98851	-0.01667	0.9168	0.037725
PE aa 36:1	0.98181	-0.026483	0.9548	0.020087
PC aa C38:6	0.9759	-0.035191	0.9548	0.020087
lysoPE a C16:1	0.70651	-0.50121	0.95852	0.018399
PS C36:1	0.9501	-0.073843	0.95926	0.018065
PC aa C36:0	1.0054	0.0078197	0.97796	0.0096776
PC ae C36:0	0.99009	-0.014375	0.97796	0.0096776
PE ae 42:2	0.92047	-0.11956	0.97796	0.0096776
PC aa C38:6	0.96221	-0.055575	0.98784	0.0053141
PE ae 42:2	0.91753	-0.12418	0.99136	0.0037708

B) Lipidomic profile siGPR17 vs neg - DID 5				
**Lipid**	**Fold change (FC)**	**log2(FC)**	**p.ajusted**	**-LOG10(p)**
PE aa 40:4	0.58499	-0.77351	0.033966	1.469
PC aa C42:5	0.50293	-0.99156	0.033966	1.469
PC aa C42:6	0.55635	-0.84593	0.036049	1.4431
PE aa 40:5	0.54963	-0.86347	0.036049	1.4431
PE aa 34:3	1.9353	0.95256	0.042192	1.3748
PC ae C32:2	2.0973	1.0685	0.048833	1.3113
PC ae C38:2	1.5238	0.60772	0.048833	1.3113

PC ae C36:2	1.5207	0.60475	0.048833	1.3113
PE aa 40:6	0.69919	-0.51625	0.048833	1.3113
PE aa 42:5	0.5765	-0.79461	0.048833	1.3113
PC ae C36:1	1.4594	0.54536	0.060947	1.215
PC aa C42:2	0.6654	-0.58772	0.060947	1.215
SM C18:1	1.8549	0.89134	0.064409	1.1911
SM (OH) C16:1	1.6952	0.76146	0.064409	1.1911
PC ae C30:1	1.6309	0.70565	0.064409	1.1911
PE aa 42:7	0.68831	-0.53888	0.064409	1.1911
PC ae C34:2	1.5716	0.65227	0.066292	1.1785
PE aa 42:6	0.66596	-0.5865	0.066292	1.1785
PC aa C40:4	0.6871	-0.5414	0.076325	1.1173
PC aa C40:5	0.65091	-0.61947	0.091207	1.04
PC ae C32:1	1.5628	0.64412	0.11468	0.94051
PC ae C38:3	1.4357	0.52176	0.11468	0.94051
PE aa 38:5	0.7727	-0.37202	0.11468	0.94051
PE aa 42:8	0.68327	-0.54948	0.11891	0.92478
PE ae 42:1	0.68279	-0.55049	0.12802	0.89272
PE ae 42:0	0.59598	-0.74668	0.12816	0.89225
PC ae C36:3	1.4526	0.53865	0.13236	0.87825
PC ae C42:0	0.56255	-0.82994	0.15319	0.81477
PC aa C42:4	0.64794	-0.62607	0.15319	0.81477
PC ae C34:3	1.436	0.52203	0.15538	0.80859
PC aa C32:1	1.3067	0.38588	0.15538	0.80859
PE aa 40:7	0.77512	-0.36751	0.15538	0.80859
PE aa 36:2	0.5832	-0.77794	0.16491	0.78275
PE ae 40:5	0.61822	-0.6938	0.1789	0.74738
PC aa C40:4	0.69663	-0.52155	0.19248	0.71561
PC aa C30:2	1.496	0.58115	0.19546	0.70894
PC aa C38:5	0.71727	-0.47941	0.21008	0.67761
PE aa 44:6	0.43106	-1.214	0.23967	0.62039
PE aa 42:4	0.63367	-0.65821	0.23967	0.62039
PC ae C42:1	0.51617	-0.95408	0.24444	0.61183
SM (OH) C14:1	1.4432	0.52923	0.30615	0.51406
PE aa 42:9	0.69083	-0.53359	0.31276	0.50478
PC aa C32:2	1.3658	0.44978	0.31276	0.50478
PC aa C38:4	0.74804	-0.41881	0.31276	0.50478
PE aa 38:4	0.75326	-0.40878	0.31276	0.50478
PE ae 40:4	0.76979	-0.37747	0.31276	0.50478
PE aa 42:10	0.62977	-0.6671	0.32494	0.4882
SM C16:1	1.4679	0.55373	0.32494	0.4882
PC aa C40:6	0.73414	-0.44587	0.32494	0.4882
PE ae 40:3	0.76847	-0.37994	0.32494	0.4882
SM C24:1	1.2621	0.33579	0.32494	0.4882
PE aa 36:1	0.81687	-0.29182	0.32494	0.4882
PE aa 40:3	0.85028	-0.234	0.32494	0.4882
PC aa C36:3	1.1242	0.16896	0.32494	0.4882
PS C38:4	1.7691	0.823	0.3308	0.48044
PC aa C36:4	0.64673	-0.62877	0.3308	0.48044
PE aa 32:1	1.504	0.5888	0.3308	0.48044
PC ae C30:0	1.4598	0.5458	0.3308	0.48044
PE ae 38:2	1.4109	0.49666	0.3308	0.48044
PC aa C38:6	0.73447	-0.44523	0.3308	0.48044

SM (OH) C22:1	1.3802	0.46492	0.33356	0.47683
SM C24:0	1.3254	0.40645	0.34211	0.46583
PC aa C36:2	1.2296	0.29824	0.35445	0.45044
lysoPC a C20:3	1.724	0.78575	0.35899	0.44491
PC aa C36:1	1.2817	0.35808	0.35947	0.44433
PI C38:5	0.73912	-0.43612	0.35986	0.44387
PC ae C42:2	0.55084	-0.8603	0.36494	0.43778
PE ae 38:4	0.78221	-0.35437	0.3682	0.43391
PE ae 36:1	1.4657	0.55159	0.37261	0.42875
lysoPE a C20:4	0.54136	-0.88535	0.37694	0.42373
SM C18:0	1.5341	0.61737	0.37694	0.42373
PC ae C40:5	0.6956	-0.52367	0.37694	0.42373
lysoPC a C16:1	1.4343	0.5203	0.37694	0.42373
lysoPC a C26:1	1.3126	0.39245	0.37694	0.42373
PC aa C32:3	1.2852	0.36199	0.37694	0.42373
PE ae 42:2	0.6718	-0.5739	0.38014	0.42005
PS C40:6	1.5092	0.59374	0.38689	0.41241
PE aa 36:8	1.5	0.58492	0.38689	0.41241
lysoPC a C18:2	1.3996	0.48503	0.38689	0.41241
PC aa C38:1	0.83393	-0.262	0.38689	0.41241
lysoPC a C18:1	1.682	0.7502	0.40139	0.39644
PC ae C40:2	1.3567	0.44007	0.40561	0.39189
PE aa 44:9	0.79611	-0.32897	0.41863	0.37817
Linoleic acid	1.3367	0.41867	0.44805	0.34867
PS C36:2	1.4578	0.54383	0.4519	0.34496
PC ae C40:3	1.1611	0.21546	0.45672	0.34035
lysoPE a C16:1	0.62203	-0.68495	0.4702	0.32772
PS C38:5	1.5119	0.59639	0.4702	0.32772
PC aa C40:2	0.80869	-0.30634	0.48098	0.31788
PE aa 38:1	1.1587	0.21249	0.50561	0.29619
GCer-d18:1/18:0	1.4482	0.53427	0.51442	0.28869
lysoPC a C16:0	1.5035	0.58836	0.5152	0.28802
PC aa C36:5	0.75511	-0.40524	0.5152	0.28802
SM C22:3	0.75511	-0.40524	0.5152	0.28802
PC ae C40:6	0.75775	-0.4002	0.5152	0.28802
PC aa C36:0	1.1915	0.25277	0.5152	0.28802
PE aa 38:6	0.93978	-0.089608	0.5152	0.28802
PE ae 40:0	0.848	-0.23787	0.53339	0.27296
PC aa C34:2	1.1314	0.17809	0.53795	0.26926
PE ae 38:3	1.1502	0.20184	0.5484	0.2609
PC aa C40:3	0.89227	-0.16444	0.5484	0.2609
PC ae C44:5	1.3724	0.45666	0.54916	0.2603
Stearic acid	0.62607	-0.6756	0.56065	0.25131
PC ae C38:1	1.2184	0.28502	0.5701	0.24405
Palmitic acid	0.66494	-0.5887	0.57211	0.24252
PC ae C40:1	0.61809	-0.69411	0.57669	0.23906
PI C38:3	1.5983	0.67652	0.58531	0.23261
Cer-d18:1/24:1	0.73956	-0.43526	0.58531	0.23261
SM C16:0	1.3071	0.38639	0.58531	0.23261
SM C26:0	0.78947	-0.34105	0.58531	0.23261
PC ae C38:4	0.81459	-0.29586	0.58531	0.23261
PE aa 36:4	0.87709	-0.1892	0.58531	0.23261
PE aa 40:8	0.87858	-0.18675	0.58531	0.23261

PE ae 38:6	0.90782	-0.13953	0.58531	0.23261
PC aa C38:3	1.0827	0.11462	0.63943	0.19421
PE aa 32:0	0.89334	-0.16272	0.64616	0.18966
PE ae 38:0	0.80833	-0.30698	0.64873	0.18793
Margaric acid	0.66459	-0.58946	0.68201	0.16621
PC ae C44:4	1.3957	0.48101	0.68201	0.16621
lysoPE a C18:2	1.0219	0.031231	0.68201	0.16621
Cer-d18:1/16:0	0.72401	-0.46593	0.68671	0.16323
PE ae 38:5	1.1582	0.21188	0.69175	0.16005
lysoPC a C28:1	1.1375	0.18591	0.69175	0.16005
PE aa 40:2	0.90099	-0.15042	0.69175	0.16005
PE aa 38:0	1.1065	0.14594	0.69433	0.15844
lysoPC a C20:4	0.77289	-0.37167	0.71735	0.14427
GCer-d18:1/16:0	1.2683	0.34288	0.71735	0.14427
PE aa 38:2	1.0864	0.11958	0.72817	0.13776
lysoPC a C18:0	1.2685	0.3431	0.73174	0.13565
PC ae C40:4	0.91863	-0.12244	0.73174	0.13565
lysoPE a C16:0	1.2068	0.27121	0.74857	0.12577
SM C20:2	0.83914	-0.25301	0.75335	0.123
PE aa 38:8	0.85287	-0.22961	0.75335	0.123
PC aa C30:0	1.0799	0.11086	0.75335	0.123
PS C38:3	0.96276	-0.054757	0.75489	0.12212
PC aa C24:0	0.7643	-0.3878	0.76265	0.11767
PS C36:1	1.2946	0.37249	0.77577	0.11027
PC aa C34:4	0.82695	-0.27413	0.77577	0.11027
PE aa 34:2	1.0879	0.12149	0.77577	0.11027
PC aa C36:6	1.086	0.11904	0.78784	0.10356
PE ae 38:1	1.0962	0.13245	0.79379	0.10029
lysoPC a C17:0	1.2242	0.29181	0.80778	0.092709
PI C38:4	0.92933	-0.10574	0.80778	0.092709
PE aa 34:4	1.2674	0.34187	0.82036	0.085994
lysoPE a C18:1	1.2512	0.3233	0.82036	0.085994
PC aa C26:0	1.1798	0.23858	0.82036	0.085994
PE aa 34:3	1.1134	0.15503	0.82036	0.085994
GCer-d18:1/24:1	1.0651	0.090928	0.82036	0.085994
PE aa 38:3	1.0587	0.08225	0.82036	0.085994
PC ae C34:0	1.0573	0.080394	0.82036	0.085994
PC ae C36:4	0.92682	-0.10963	0.82036	0.085994
PC ae C38:5	0.86418	-0.2106	0.82036	0.085994
Nervonic acid	0.83533	-0.25958	0.82036	0.085994
Arachidonic acid	0.77426	-0.36911	0.82036	0.085994
PC ae C44:3	0.71795	-0.47805	0.82036	0.085994
lysoPE a C18:0	0.68534	-0.5451	0.82036	0.085994
PS C40:5	0.62687	-0.67375	0.82036	0.085994
PE aa 36:6	1.0289	0.041166	0.82117	0.085569
lysoPC a C24:0	1.0128	0.018418	0.82199	0.085132
Cer-d18:1/18:0	0.75849	-0.39879	0.82199	0.085132
PC ae C34:1	1.0646	0.090269	0.8257	0.08318
PE aa 40:1	0.84858	-0.23688	0.8257	0.08318
PE aa 32:2	0.98082	-0.027946	0.82886	0.081521
PC aa C38:0	0.92846	-0.10708	0.83273	0.079497
PC aa C34:1	1.0181	0.025818	0.84325	0.074044
lysoPC a C28:0	0.94371	-0.083578	0.84325	0.074044

PE aa 40:10	0.82632	-0.27523	0.84325	0.074044
SM (OH) C24:1	0.6314	-0.66338	0.84325	0.074044
Oleic acid	0.99818	-0.002633	0.84471	0.073291
PE aa 36:5	0.98065	-0.028187	0.84471	0.073291
PS C40:7	1.0662	0.092454	0.85547	0.067794
PE ae 40:1	1.0657	0.091792	0.85547	0.067794
PE aa 40:9	1.0392	0.05552	0.85547	0.067794
PC aa C32:0	1.0355	0.05029	0.85547	0.067794
PC ae C36:0	1.0071	0.010172	0.85547	0.067794
PE aa 34:0	0.91348	-0.13055	0.85547	0.067794
PC ae C38:0	0.85915	-0.21902	0.85547	0.067794
PC ae C36:5	1.1024	0.14066	0.8727	0.059135
PE aa 34:1	0.91328	-0.13087	0.88491	0.053101
lysoPC a C14:0	1.3115	0.39118	0.89596	0.047713
PE aa 38:7	1.042	0.059339	0.91407	0.03902
PE aa 36:3	1.0256	0.036453	0.91407	0.03902
PC ae C44:6	0.90256	-0.14791	0.91407	0.03902
PC aa C28:1	1.145	0.19541	0.95709	0.019046
PE ae 40:7	0.99316	-0.009903	0.95709	0.019046
PC aa C34:3	0.99559	-0.006379	0.96072	0.017405
lysoPC a C26:0	1.0687	0.095901	0.97969	0.0089092
PE ae 34:0	0.9798	-0.029443	0.97969	0.0089092
lysoPE a C14:0	0.97539	-0.035956	0.97969	0.0089092
PC ae C38:6	0.94888	-0.075695	0.97969	0.0089092
PC ae C30:2	0.90037	-0.15141	0.97969	0.0089092
SM C26:1	0.99244	-0.010953	0.98842	0.0050592
